# CircNEIL3 regulatory loop promotes pancreatic ductal adenocarcinoma progression via miRNA sponging and A-to-I RNA-editing

**DOI:** 10.1186/s12943-021-01333-7

**Published:** 2021-03-09

**Authors:** Peng Shen, Taoyue Yang, Qun Chen, Hao Yuan, Pengfei Wu, Baobao Cai, Lingdong Meng, Xumin Huang, Jiaye Liu, Yihan Zhang, Weikang Hu, Yi Miao, Zipeng Lu, Kuirong Jiang

**Affiliations:** 1 Pancreas Center, the First Affiliated Hospital of Nanjing Medical University, Nanjing, China; 2 Pancreas Institute, Nanjing Medical University, Nanjing, China; 3 Nanjing Medical University, Nanjing, China

**Keywords:** Pancreatic ductal adenocarcinoma, circNEIL3, miR-432-5p, ADAR1, GLI1, RNA editing, Cell cycle, EMT

## Abstract

**Background:**

A growing number of studies have focused on investigating circRNAs as crucial regulators in the progression of multiple cancer types. Nevertheless, the biological effects and underlying mechanisms of circRNAs in pancreatic ductal adenocarcinoma (PDAC) remain unclear.

**Methods:**

Differentially expressed circRNAs between cancerous tissue and adjacent normal tissues were identified by RNA sequencing in PDAC. Subsequently, in vitro and in vivo functional experiments were performed to investigate the functional roles of circNEIL3 in PDAC tumour growth and metastasis. Furthermore, RNA pull-down, dual-luciferase reporter assays, RNA immunoprecipitation (RIP) assays, fluorescent in situ hybridization (FISH) and Sanger sequencing assays were performed to examine the circular interaction among circNEIL3, miR-432-5p and adenosine deaminases acting on RNA 1 (ADAR1).

**Results:**

CircNEIL3 was upregulated in PDAC and promoted the progression of PDAC cells both in vitro and in vivo. Mechanistically, circNEIL3 was shown to regulate the expression of ADAR1 by sponging miR-432-5p to induce RNA editing of glioma-associated oncogene 1 (GLI1), ultimately influencing cell cycle progression and promoting epithelial-to-mesenchymal transition (EMT) in PDAC cells. Moreover, we discovered that the circNEIL3/miR-432-5p/ADAR1 axis was correlated with the PDAC clinical stage and overall survival of PDAC patients, while ADAR1 may reduce the biogenesis of circNEIL3.

**Conclusions:**

Our findings reveal that circNEIL3 facilitates the proliferation and metastasis of PDAC through the circNEIL3/miR-432-5p/ADAR1/GLI1/cell cycle and EMT axis and that its expression is regulated by ADAR1 through a negative feedback loop. Therefore, circNEIL3 may serve as a prognostic marker and a therapeutic target for PDAC.

**Supplementary Information:**

The online version contains supplementary material available at 10.1186/s12943-021-01333-7.

## Introduction

Pancreatic cancer is one of the most malignant tumours and has a 5-year survival rate of less than 9% [1]. Early metastasis and local invasion combined with a lack of therapeutic efficacy of cytotoxic, targeted, and immune-based therapeutics, makes PDAC the 3rd leading cause of cancer-related death in the United States [2]. Furthermore, even for the small subset of patients diagnosed with a localized, resectable tumour, the prognosis remains poor, with only 37% of patients surviving 5 years following surgery [3]. Thus, there is an urgent need to identify potential molecular targets or genetic regulatory networks of PDAC.

Circular RNAs (circRNAs) are noncoding RNAs originating from the back-splicing of pre-mRNA transcripts to form a covalently closed continuous loop [4, 5]. Compared to their linear counterparts, circRNAs exhibit higher stability due to the loop structure being resistant to RNase R [6]. Therefore, the biological function of circRNAs has been widely studied, especially with respect to tumour progression, metastasis, apoptosis, and autophagy [7]. MiRNAs are also noncoding RNAs that can directly bind to the 3’UTRs of target mRNAs to form an RNA-induced silencing complex (RISC) and regulate gene expression at the posttranscriptional level [8, 9]. Emerging evidence has shown that circRNAs primarily function as microRNA (miRNA) sponges to regulate downstream target genes [8, 10]. In addition, some circRNAs have been shown to interact with RNA binding proteins (RBPs) or function as templates for protein translation [11, 12]. However, the underlying mechanisms of how circRNAs regulate PDAC progression and metastasis remain elusive, which enhances the significance of the findings of the present study.

RNA editing contributes to RNA mutations [13]. Key enzymes involved in this process are the adenosine deaminases acting on RNA (ADAR) family of enzymes, including ADAR1 and ADAR2, which perform adenosine-to-inosine (A-to-I) editing of double-stranded RNA (dsRNA) [14]. Recent studies have shown that deleting ADAR1 can increase cell vulnerability and render tumour cells more sensitive to immunotherapy, implicating ADAR1 as a potential immuno-oncology target [15–17]. ADAR1 has been reported to enhance Alu-dependent editing and transcriptional activity of GLI1 [18]. As a terminal factor of the Hedgehog pathway, GLI1 has been reported to function as transcriptional activator involved in cell cycle progression and EMT [19, 20]. However, the function and mechanism of ADAR1 in PDAC remain elusive.

In our present study, through RNA-seq and RT-qPCR analyses, we identified an oncogenic circRNA generated from the linear counterpart of the NEIL3 gene, termed circNEIL3. We demonstrated that circNEIL3 was notably upregulated in PDAC tissues and cells and induced proliferative and metastatic phenotypes in vitro and in vivo. Significantly, we revealed that circNEIL3 can sponge miR-432-5p to upregulate ADAR1 expression and promote PDAC proliferation and metastasis. Clinically, circNEIL3, miR-432-5p and ADAR1 dysregulation were shown to be related to the prognosis and clinicopathological characteristics of patients with PDAC. Collectively, our results revealed that the circNEIL3/miR-432-5p/ADAR1/GLI1 axis has a considerable role in PDAC proliferation and metastasis, identifying circNEIL3 as a potential biomarker and therapeutic target for PDAC.

## Methods

### RNA-seq

Total RNA was extracted from three pairs of frozen (in liquid nitrogen) PDAC tissues and adjacent normal tissues using TRIzol Reagent (Invitrogen, CA, USA) and then treated using a RiboMinus Eukaryote kit (Qiagen, Valencia, CA) to remove ribosomal RNA before generating the RNA-seq library. Next, the RNA-seq library was deep sequenced with an Illumina HiSeq 2000 instrument (Illumina, San Diego, CA). The RNA-seq FASTQ reads were first aligned to the human reference genome (hg38/GRCh38) using TopHat2. The sequences that aligned contiguously and along their full length to the genome were discarded, and the remaining reads were used to identify circRNAs. We applied the spliced reads per billion mapping (SRPBM) approach to normalize the counts of reads mapped across an identified back-splice junction. The sequencing results of all differentially expressed circRNAs are shown in Tab. S1, and the raw data is available in the NCBI database with the access number PRJNA695439.

### Patients and clinical specimens

Pancreatic tissue specimens, including tumour and adjacent normal tissues, were obtained from 104 patients receiving a pancreaticoduodenectomy for PDAC at the First Affiliated Hospital of Nanjing Medical University from Jul. 2014 to Dec. 2018. The 104 patients were followed regularly until 11 September 2020. None of the patients had received chemotherapy or radiotherapy before surgery. All patients provided written informed consent that was approved by the Hospital Ethics Committee before specimen collection. The tissue specimens were collected during the surgery and immediately (within 3 min) cut into two sections. One section was frozen in liquid nitrogen, while the other was fixed in 4% formalin and embedded in paraffin 24 h later for confirmation of the pathological diagnosis. All of the cancer and adjacent tissues were diagnosed by two pathologists independently. The overall survival of patients was defined as the time between surgery and death. None of the 104 selected patients died within 1 month after surgery.

### Cell culture

Four human PDAC cell lines (BxPC-3, MiaPaca-2, CFPAC-1 and PANC-1) and human pancreatic ductal epithelial (HPNE) cells were purchased from the Cell Bank of Type Culture Collection of the Chinese Academy of Sciences in Shanghai, China. The cells were cultured in Dulbecco’s modified Eagle’s medium (Life Technologies) supplemented with 10% foetal bovine serum (Wisent, Montreal, QC, Canada), 10 mM HEPES (Sigma, St Louis, MO), 2 mM L-glutamine (Sigma), 1 mM sodium pyruvate (Sigma), 100 U/ml of penicillin (Life Technologies) and 100 μg/ml streptomycin (Life Technologies) at 37 °C in a humidified atmosphere containing 95% air and 5% CO_2_.

### Plasmid construction, siRNA interference and lentiviral infection

To construct circNEIL3-overexpressing plasmids, human circNEIL3 cDNA was synthesized and cloned into the vector pGL3-cir by Obio Technology Corp., Ltd. (Shanghai, China), with the empty plasmid used as a control. CircNEIL3 siRNA sequences were synthesized by GenePharma (Shanghai, China), and a scrambled siRNA was synthesized as a negative control. Transfection was performed using Lipofectamine 3000 (Invitrogen) according to the manufacturer’s instructions. Total RNA was collected 48 h after transfection.

The circNEIL3-knockdown lentivirus was constructed by Corues Biotechnology Corp., Ltd. (Nanjing, China). Si-circNEIL3–2 was subcloned into the vector pLV3ltr-Luc-Puro-U6-siRNA (circNEIL3) and was verified by sequencing. The supernatant of the cultured 293 T cells was collected to infect MiaPaca-2 and CFPAC-1 cells. Stable cell lines were selected by culturing in medium containing 5 μg/ml puromycin (Sigma). CircNEIL3 expression was confirmed by RT-qPCR.

The miR-432-5p mimics and inhibitor, ADAR1-overexpressing lentivirus and ADAR1 shRNAs were synthesized by GenePharma (Shanghai, China) and transfected as described above. All the sequences used in the present study are listed in Tab. S2.

### RNA extraction and RT-qPCR

Total RNA was extracted from cell and tissue samples using TRIzol Reagent (Life Technologies, Carlsbad, CA, USA) according to the manufacturer’s instructions. After spectrophotometric quantification, 1 μg of total RNA in a final volume of 20 μl was used for reverse transcription (RT) with an iScript cDNA Synthesis Kit (Bio-Rad, Hercules, CA, USA) following the manufacturer’s instructions. According to the manufacturer’s protocol, total cDNA was then used for RT-qPCR with the TaqMan Gene Expression Assay (Thermo Fisher Scientific, Rockford, IL, USA) in a StepOne Plus Real-time PCR System (Thermo Fisher Scientific). The expression of human 18S rRNA or β-actin genes was used as a control to calibrate the original concentration of tissue or cell mRNA, respectively. Target gene expression was calculated using the 2^-ΔΔCT^ method. Each quantitative PCR assay was performed in triplicate and independently repeated three times. The sequences of the primers used in the present study are listed in Tab. S2.

### Nuclear and cytoplasmic extraction

Nuclear and cytoplasmic fractions were isolated using the reagents in a PARIS™ kit (AM1556, Thermo Fisher Scientific, Waltham, USA). Briefly, CFPAC-1 and MiaPaca-2 cells were lysed in Cell Fraction Buffer on ice for 10 min. Subsequently, after centrifugation at 500 *g* for 3 min at 4 °C, the supernatant was collected as the cytoplasmic fraction. Then, the pelleted nuclei were washed with Cell Fraction Buffer and used as the nuclear fraction.

### RNase R/Actinomycin D

Total RNA (2 μg) was incubated with 3 U/μg RNase R (Epicentre Technologies, Madison, WI, USA) for 15 min at 37 °C. CFPAC-1 and MiaPaca-2 cells were transferred to six-well plates and treated with 5 μg/ml actinomycin D when the number of cells reached 900,000, with samples collected at the indicated time points. The expression of circNEIL3 and the linear counterpart mRNA NEIL3 was analysed by RT-qPCR.

### RNA fluorescence in situ hybridization

Cy3-labelled circNEIL3 probes and fluorescein amidite (FAM)-labelled miR-432-5p probes were designed and synthesized by RiboBio. A fluorescence in situ hybridization (FISH) kit (RiboBio) was used to detect the probe signals in CFPAC-1 and MiaPaca-2 cells according to the manufacturer’s instructions. Nuclei were stained with 4,6-diamidino-2-phenylindole (DAPI). All images were acquired with an LSM880 NLO (2 + 1 with BIG) confocal microscope system (Carl Zeiss).

### Functional experiment

#### CCK-8 assay

We used a CCK-8 assay kit (Dojindo, Japan) to assess cell proliferation. Cells were seeded in 96-well plates at 1.0 × 10^3^ cells/well. Subsequently, each day of the following 5 days, 10 μl of CCK-8 reagent and 100 μl of complete medium were mixed and added to each well. After incubating for 2 h at 37 °C away from light, the absorbance of each well at 450 nm was measured with a microplate reader. Each sample was evaluated with five replicate wells, and the assay was independently repeated three times.

#### Clone formation assay

Cells were seeded in six-well plates (800 cells/well) and cultured in complete medium supplemented with 10% foetal bovine serum for 2 weeks. Subsequently, the cells were stained with 0.1% crystal violet (Beyotime) for 30 min and then washed once with PBS. The colonies were then counted if their diameter was greater than 1 mm.

#### 5-Ethynyl-20- deoxyuridine (EdU) assay

The EdU assay was performed to assess the cell proliferation using an EdU Proliferation Kit (Beyotime). PDAC cells were plated in 48-well plates and cultured for 24 h, after which they were incubated with a 50 mM EdU solution for 2 h and then fixed in 4% paraformaldehyde. According to the manufacturer’s protocol, the cells were then permeabilized with 0.3% Triton for 10 min and then sequentially stained with Alexa Fluor 555 azide and Hoechst 33342. Subsequently, the EdU-treated cells were imaged and counted under an Olympus FSX100 microscope (Olympus, Tokyo, Japan).

#### Wound healing assay

Wound healing assays were performed to evaluate the migration ability of PDAC cells. Forty-eight hours after seeding cells into six-well plates (8 × 10^5^ cells/well), cell monolayers were scratched with a 200-μl pipette tip to produce lesions of a consistent length and then cultured in basal medium. After washing the cells with PBS to remove cellular fragments, each wound was imaged at 0, 24 and 48 h by inversion microscopy (Olympus, Japan). Cell migration was quantified by measuring the relative wound areas using ImageJ.

#### Transwell assay

Approximately 4 × 10^4^ MiaPaca-2 and CFPAC-1 cells were uniformly seeded into the upper layer of each Transwell membrane, and culture medium (750 μl) containing 10% foetal bovine serum was used as a chemoattractant to induce cell migration to the other side. After incubating at 37 °C under an atmosphere with 5% CO_2_ for 24 h, the cells above the membrane were gently wiped off using cotton-tipped swabs, while the cells that passed through the membrane were stained with 0.1% crystal violet for 30 min to assess cell migration. Finally, representative images from five random views were obtained under a microscope. Matrigel (BD Bioscience Pharmingen) was spread on the upper layer to assess cell invasion according to the manufacturer’s protocol, and the remaining procedure following the steps described above.

#### Flow cytometry assay

Flow cytometry analysis was performed using a Cell Cycle Analysis Kit (Beyotime, Shanghai, China) to evaluate the cell cycle according to the manufacturer’s instructions. After 48 h of transfection, the cells were digested and washed with PBS twice and then fixed in 75% alcohol overnight at − 20 °C. Subsequently, the fixed cells were washed three times and then stained with propidium iodide (PI) staining buffer at room temperature for 30 min in the dark, after which the cell cycle was analysed by flow cytometry (LSR, BD Biosciences).

### Biotin-labelled RNA pull-down

MiaPaca-2 and CFPAC-1 cells were harvested and lysed, and C-1 magnetic beads were incubated with the circNEIL3 probe (Life Technologies) at 25 °C for 2 h to generate probe-coated beads. The cell lysates were then incubated with circNEIL3 or an oligo probe at 4 °C. The RNA complexes bound to the beads were then eluted and extracted with an RNeasy Mini Kit (Qiagen) for RT-qPCR analysis. The biotinylated-circNEIL3 probe was designed and synthesized by RiboBio (Guangzhou, China).

### Dual-luciferase assay

Wild-type and mutant (mut-circNEIL3 or mut-ADAR1) cirCNEIL3 and ADAR1 fragments were constructed and inserted downstream of the luciferase reporter gene in the reporter plasmid pRL-SV40 (GenePharma, Shanghai, China). PDAC cells were seeded in 24-well plates and grown to 30% confluence 24 h before being transfected with the reporter plasmid using Lipofectamine 3000. Cells were also cotransfected with different combinations of plasmids harbouring the 3′-untranslated region (3′-UTR) of assayed genes (500 ng) and miRNA mimics or the negative control (NC; 10 nM final concentration). After 48 h, the activities of both firefly luciferase (LUC) and Renilla luciferase (RLUC) were measured with a Dual-Luciferase Reporter System Kit (E1910, Promega, USA).

### RNA immunoprecipitation (RIP)

RNA immunoprecipitation (RIP) assays were performed using a Magna RIP RNA-Binding Protein Immunoprecipitation Kit (Millipore, Billerica, MA, USA). Approximately 1 × 10^7^ PDAC cells were lysed in 1 ml of RIP lysis buffer supplemented with protease and RNase inhibitors. The cell lysates were then incubated with IgG, anti-AgO2 or anti-ADAR1 antibody-coated beads (Millipore) and rotated at 4 °C overnight. The immunoprecipitated RNAs were subsequently extracted with an RNeasy MinElute Cleanup Kit (Qiagen, Valencia, CA, USA) after treatment with proteinase K buffer and reverse transcribed to cDNA. Subsequently, the mRNA levels of the assayed genes were measured by RT-qPCR.

### Western blot analysis

Proteins were extracted from stably transfected cells with RIPA buffer supplemented with PMSF. A DC Protein Assay Kit (Bio-Rad) was used to quantify the protein concentrations in the cell lysates. Then, the proteins were separated via electrophoresis using SDS-containing polyacrylamide gels and then transferred to polyvinylidene fluoride (PVDF) membranes (Millipore, Billerica, MA, USA). After blocking the membranes with 5% nonfat dry milk in 0.1% Tween (TBST) buffer at room temperature for 2 h, the membranes were incubated at 4 °C overnight with the appropriate primary antibody. Subsequently, the membranes were washed 3 times with TBST buffer for 15 min. Then, the membranes were incubated with a corresponding HRP-labelled secondary antibody for 2 h at room temperature, after which they were washed 3 times with TBST buffer. Finally, the western blot signals were visualized using an enhanced chemiluminescence detection system with Chemiluminescence HRP Substrate (Millipore, WBKL0100). All of the primary and secondary antibodies used in this study are listed in Tab. S3.

### Animal experiments

Four-week-old male nude mice (BALB/c) were purchased from the Animal Center of Nanjing Medical University (Nanjing, China). Ten mice per group were used to construct subcutaneous tumour formation models, and cell suspensions (0.1 ml) were prepared containing 1 × 10^6^ stable cells (CFPAC-1-circNEIL3 KD, CFPAC-1-circNEIL3-KDNC, MiaPaca-2-circNEIL3 KD, and MiaPaca-2-circNEIL3 KDNC). Then, the suspensions were subcutaneously injected into the armpits of the mouse limbs. When a tumour became macroscopic, it was measured with callipers every 5 days, and its bulk was calculated according to the following formula: volume = (width^2^ × length)/2. The sample size was not predetermined for these experiments. All experiments were performed following the relevant institutional and national guidelines and regulations.

For metastasis studies, cells (1 × 10^5^) were injected into the tail veins of mice (six mice per group). Lung metastasis was monitored using a Xenogen IVIS Spectrum Imaging System (PerkinElmer, USA). After 8 weeks, the lungs and livers of mice were excised under anaesthesia, and the numbers of macroscopically visible lung and liver metastatic nodules were counted and validated using haematoxylin and eosin (HE)-stained sections by microscopy.

### Immunohistochemistry (IHC)

Immunohistochemistry (IHC) was performed according to the manufacturer’s protocol. After the xenografts and PDAC tissues were deparaffinized with xylene and rehydrated with ethanol, the samples were incubated with 3% H_2_O_2_ for 5 min to block endogenous peroxidase activity. Then, antigen retrieval was performed by incubating the samples with sodium citrate buffer (pH 6.0) for 20 min at 95 °C, after which the samples were blocked with 5% normal goat serum for 10 min at 20 °C. Subsequently, the sections were incubated with polyclonal antibodies against ADAR1 and Ki-67 at 4 °C overnight and then incubated with secondary antibodies. The validity and signal intensity of the tissue sections were assessed by two independent pathologists. The tissue sections were scanned, and the protein levels were calculated as positive cells/total cells by Halo v3.0.311.314. All of the primary and secondary antibodies used in this study are listed in Tab. S3.

### Sanger sequencing

Total RNA was extracted by TRIzol and subjected to cDNA synthesis using an iScript cDNA Synthesis Kit (Bio-Rad, Hercules, CA, USA). The cDNA was used as a template to amplify sequences containing RNA-seq-identified ADAR1-dependent putative editing sites. Individual clones were sequenced using Applied Biosystems BigDye terminator mix version 3.1. By contrast, A-to-I calls made from RNA-seq but not verified by Sanger sequencing as A-to-G are referred to as false positives. The heights of different base peaks from the Sanger sequence were measured by ImageJ software.

### Statistical analysis

Quantitative data are presented as the means ± SDs. Differences between the means of two samples were analysed by Student’s t-test, while one-way ANOVA was used for multiple groups. Correlations between circNEIL3, miR-432-5p and ADAR1 expression and various clinicopathological or serological variables were analysed by the Mann-Whitney U test. Survival distributions and overall survival (OS) rates were determined using the Kaplan-Meier method, and the significance of differences between survival rates was calculated by the log-rank test. The above statistical analysis was performed using GraphPad Prism 8.0 (GraphPad Software, La Jolla, USA). While SPSS 20.0 (IBM, SPSS, Chicago, IL, USA) was applied in the univariate and multivariate Cox proportional hazards model, which was used to estimate the adjusted hazard ratios and 95% confidence intervals, as well as identify independent prognostic factors.

## Results

### CircNEIL3 is identified and generated from exons 8 and 9 of NEIL3 by back-splicing

**Fig. 1:**
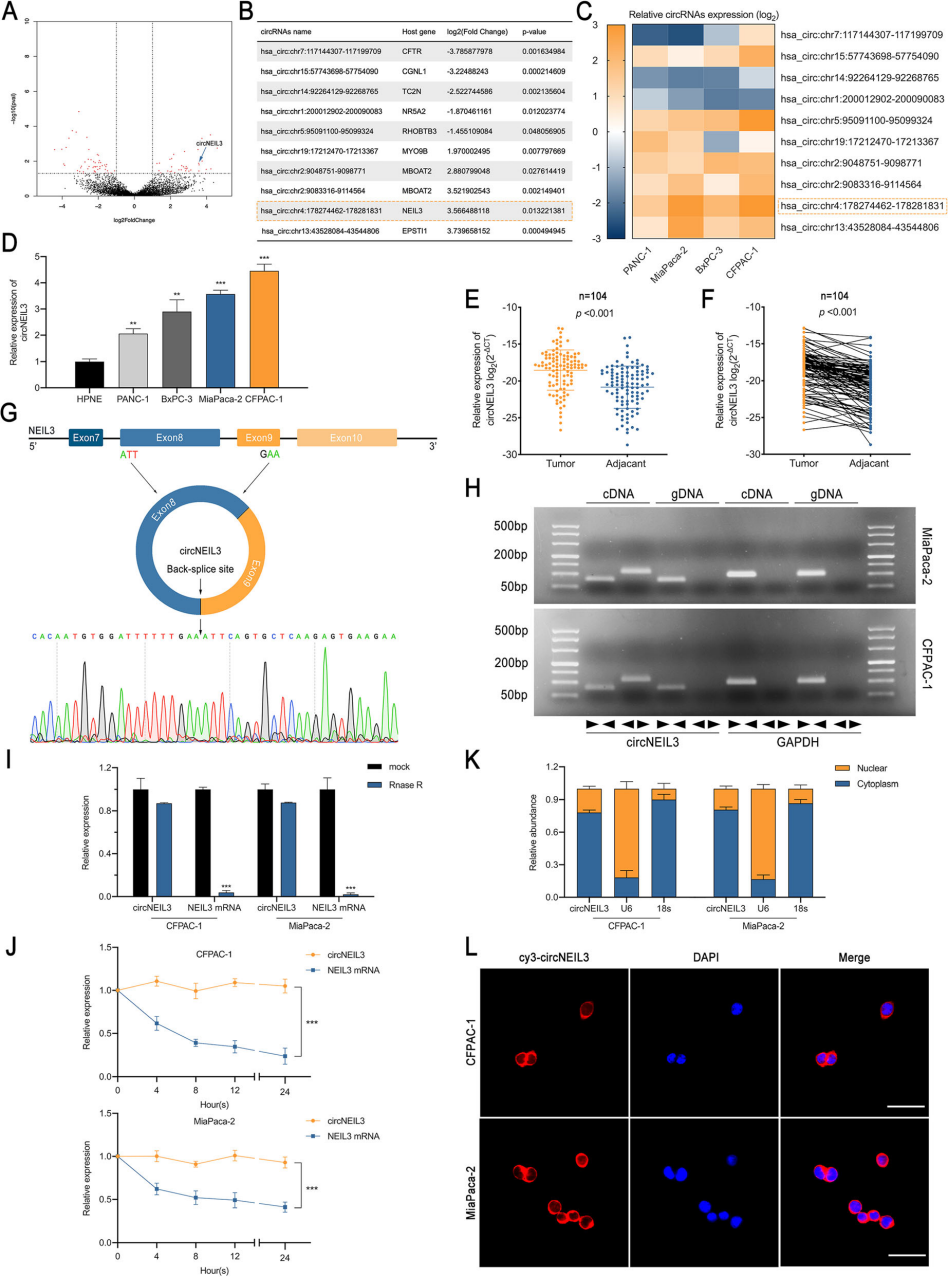
Identification and characterization of circNEIL3 in PDAC cells and tissues. **a**. Volcano plots showing 79 upregulated and 124 downregulated circRNAs in PDAC tissue relative to matched normal tissue. **b**. Basic information for the ten most dysregulated circRNAs. **c.** RT-qPCR analysis of the ten most dysregulated circRNAs in PANC-1, MiaPaca-2, BxPC-3 and CFPAC-1 cells compared to HPNE cells. **d**. Relative circNEIL3 expression in cell lines was determined by RT-qPCR. **e-f**. Relative circNEIL3 expression in PDAC tissues (tumour) and adjacent nontumour tissues (adjacent) was detected by RT-qPCR (*n* = 104). **g**. Schematic illustration of the genomic location and back splicing of circNEIL3, with the splicing site validated by Sanger sequencing. **h**. PCR and agarose gel electrophoresis analysis were performed to detect the presence of circNEIL3 and NEIL3 in cDNA and gDNA samples from PDAC cells using divergent and convergent primers. **i**. CircNEIL3 and linear NEIL3 expression in PDAC cells was detected after RNase treatment R compared to the mock treatment. **j**. Actinomycin D treatment was used to evaluate the stability of circNEIL3 and NEIL3 mRNA in PDAC cells. **k**. Nuclear-cytoplasmic fractionation assay results indicated that circNEIL3 was primarily localized in the cytoplasm of PDAC cells. The 18S rRNA and U6 genes were used as cytoplasmic and nuclear controls, respectively. **l**. FISH results showed the cellular localization of circNIEL3. The circNIEL3 probe was labeled with Cy3 (red), while nuclei were stained with DAPI (blue). The samples were imaged at 1000× magnification. Scale bar = 10 μm. All data are presented as the means ± SD of three independent experiments. *p < 0.05, **p < 0.01, ***p < 0.001

To identify circRNAs that are crucial to PDAC progression, RNA-seq was performed for 3 pairs of PDAC and matched normal pancreas tissues. As a result, 6980, 3486, 7247, 4319, 5832 and 4197 circRNAs were predicted in the three normal and three corresponding PDAC tissue samples, respectively. With the cut-off criteria of fold-change > 1 and *P*-value < 0.05, 203 differentially expressed circRNAs were identified, 79 of which were upregulated and 124 of which were downregulated (Fig. 1a). Among these circRNAs, the top 10 dysregulated circRNAs were selected for further analysis based on their fold change (Fig. 1b). To rectify the “Type I” RNA-seq error, a heat map was used to visualize the variation in the expression of these circRNAs in four PDAC cell lines compared to the normal pancreas cell line HPNE (Fig. 1c-d). CFPAC-1 and MiaPaca-2 PDAC cells were selected for further study due to their relative high expression. In general, circNEIL3 (chr4:178274462–178,281,831) was a significantly upregulated circRNA in both RNA-seq and PDAC cell lines. Furthermore, we detected higher circNEIL3 expression in 104 PDAC samples than in their paired the adjacent normal tissue samples via RT-qPCR, which was also consistent with the RNA-seq data (Fig. 1e-f). Based on the circBase annotation, circNEIL3 is derived from exons 8 and 9 of the NEIL3 gene and has a length of 596 bp [21]. Sanger sequencing was performed to validate its back-splicing using the RT-PCR product of circNEIL3 (Fig. 1g). To further confirm the circular form of circNEIL3, we designed divergent and convergent primers to amplify the circular and linear forms of NEIL3, respectively. The agarose gel electrophoresis analysis of the RT-qPCR products showed that circNEIL3 was only amplified from cDNA, ruling out the possibility of genomic rearrangements and trans-splicing (Fig. 1h). Moreover, after treatment with RNase R and actinomycin D, circNEIL3 was more stable than linear NEIL3 (Fig. 1i-j). In addition, the nuclear-cytoplasmic fractionation and FISH assay results revealed that circNEIL3 was primarily located in the cytoplasm (Fig. 1k-l). Taken together, these results identified circNEIL3 as an upregulated and highly stable circRNA localized in the cytoplasm of PDAC cell lines.

### CircNEIL3 promotes the proliferation, migration and invasion of PDAC cells in vitro

**Fig. 2:**
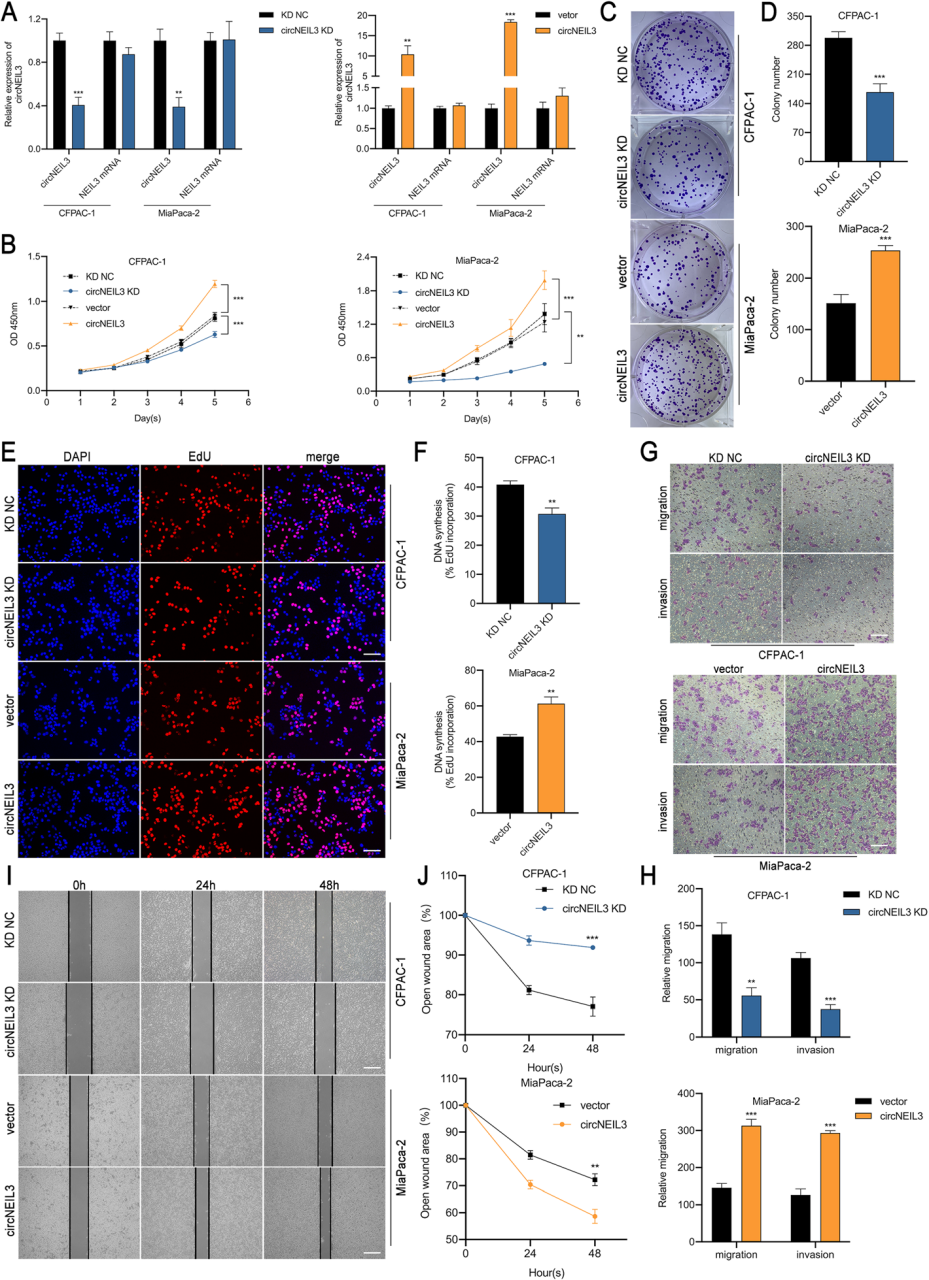
CircNEIL3 promotes the proliferation, migration and invasion of PDAC cells in vitro. **a** RT-qPCR analysis of circNEIL3 and NEIL3 mRNA expression in CFPAC-1 and MiaPaca-2 cells transfected with a lentivirus and circNEIL3 plasmid. **b.** The growth curves of cells were evaluated by CCK-8 assays after knocking down and overexpressing circNEIL3 in CFPAC-1 and MiaPaca-2 cells. **c-d.** Colony formation assays were performed to evaluate cell proliferation. **e-f.** EdU assays of PDAC cells was performed to evaluate cell proliferation. The samples were imaged at 200× magnification. Scale bar = 50 μm. **g-h**. Transwell assays were performed to assess the migration and invasion abilities of PDAC cells. The samples were imaged at 100× magnification. Scale bar = 100 μm. **i-j.** Cell migration was assessed using a wound healing assay. The samples were imaged at 100× magnification. Scale bar = 100 μm. All data are presented as the means ± SD of three independent experiments. *p < 0.05, **p < 0.01, ***p < 0.001

To assess the biological function of circNEIL3 in PDAC cells, three short interfering RNAs targeting the back-splice site of circNEIL3 were constructed to specifically downregulate circNEIL3 expression, and the one that provided the most significant downregulation was cloned into a lentivirus for further study (Fig. S1a). In addition, cell lines were also transfected with the circNEIL3 plasmid to overexpress circNEIL3 without affecting NEIL3 expression. The efficiency and specificity of circNEIL3 knockdown and overexpression in CFPAC-1 and MiaPaca-2 cells were verified by RT-qPCR (Fig. 2a). CCK-8, colony formation and EdU assays were carried out to evaluate cell proliferation, and the results showed that circNEIL3 silencing notably inhibited the proliferation of CFPAC-1 and MiaPaca-2 cells, while circNEIL3 upregulation increased cell growth (Fig. 2b-f and Fig. S1b-e). In transwell and wound healing assays, we found that circNEIL3 knockdown reduced the migration and invasion of PDAC cells, while circNEIL3 overexpression had the opposite effect. Taken together, these data suggest that circNEIL3 exerts an oncogenic role in PDAC cells (Fig. 2g-j and Fig. S1f-i).

### CircNEIL3 facilitates the tumorigenesis and metastasis of PDAC cells in vivo

**Fig. 3:**
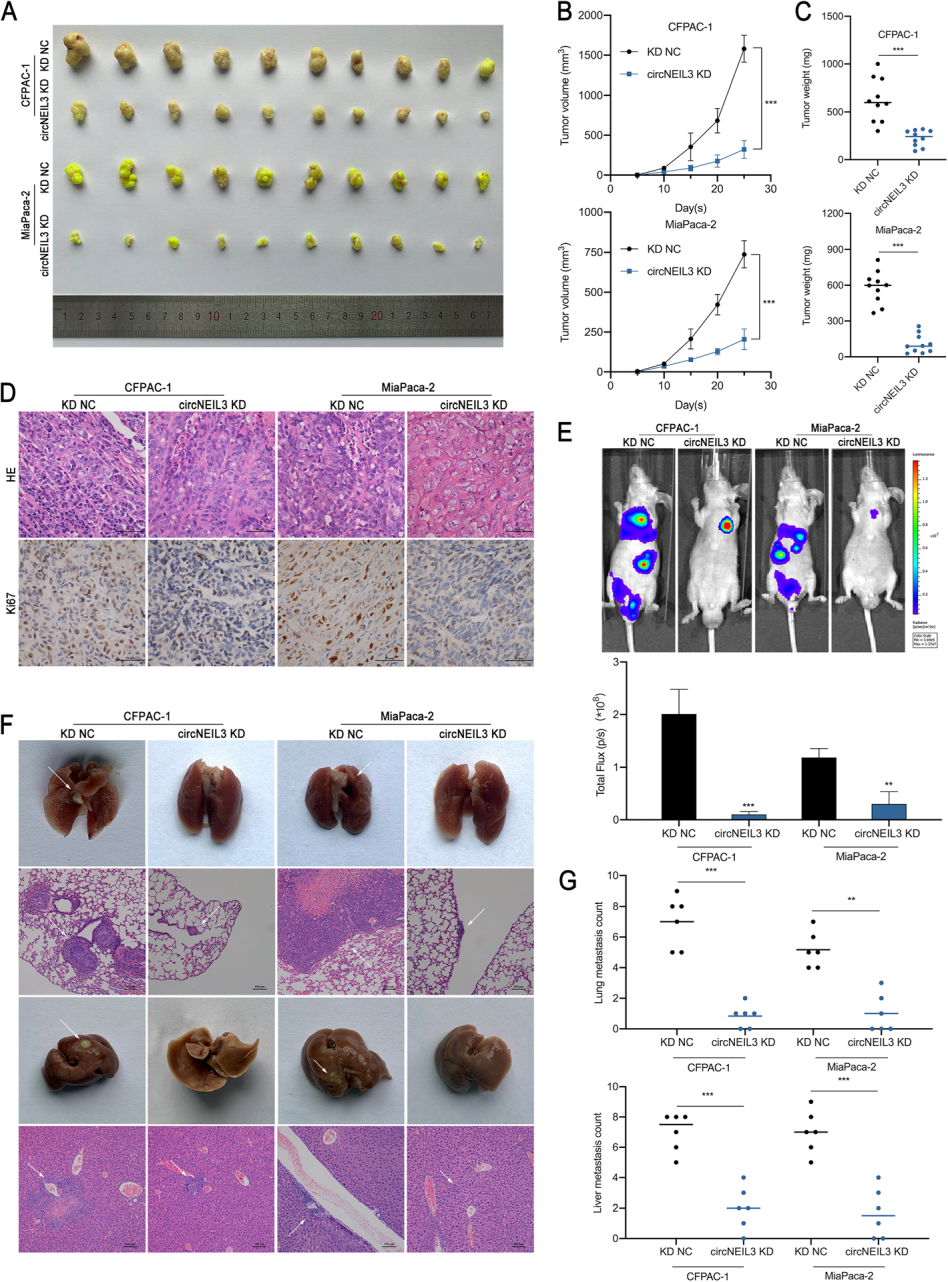
CircNEIL3 facilitates the tumorigenesis and metastasis of PDAC cells in vivo. **a**. Representative images of subcutaneous xenograft tumours (n = 10 for each group). **b**. Growth curves of tumour volumes, which were measured every five days. **c**. Tumour weights were analyzed. **d**. HE and IHC staining of xenograft tumours. The protein levels of Ki67 were analyzed based on IHC staining. The samples were imaged at 400× magnification. Scale bar = 50 μm. **e**. Representative images and analysis of luminescence intensity in tail vein-injected mouse models (*n* = 6 for each group). **f**. Representative images and HE staining of metastatic tumours in the lungs and livers of mice. The HE staining samples were imaged at 100× magnification. Scale bar = 100 μm. **g**. The number of lung and liver metastatic tumours were determined. All data are presented as the means ± SD. *p < 0.05, **p < 0.01, ***p < 0.001

To evaluate the contribution of circNEIL3 to PDAC tumours in vivo, CFPAC-1 and MiaPaca-2 cells stably transfected with LV-si-circNEIL3 and respective negative control were subcutaneously injected into male nude mice. The results showed that circNEIL3 knockdown inhibited tumour growth in both cell lines (Fig. 3a). Lower tumour weights and volumes were observed in the circNEIL3 KD group compared to the NC group (Fig. 3b-c). In addition, IHC staining revealed decreased Ki-67 levels in tumour cells after circNEIL3 knockdown (Fig. 3d). Furthermore, to determine whether circNEIL3 can promote PDAC metastasis in vivo, luciferase-labelled CFPAC-1 and MiaPaca-2 constructs were injected into the tail veins of male nude mice. The fluorescence intensity and proportion in the lung and abdomen were significantly lower in the circNEIL3 KD group than in the control group (Fig. 3e). Moreover, circNEIL3 knockdown notably decreased the number of metastatic nodules in the lung and liver (Fig. 3f-g). These results are consistent with the in vitro findings, suggesting that circNEIL3 can promote PDAC tumorigenesis and metastasis of in vivo.

### CircNEIL3 functions as a sponge of miR-432-5p

**Fig. 4:**
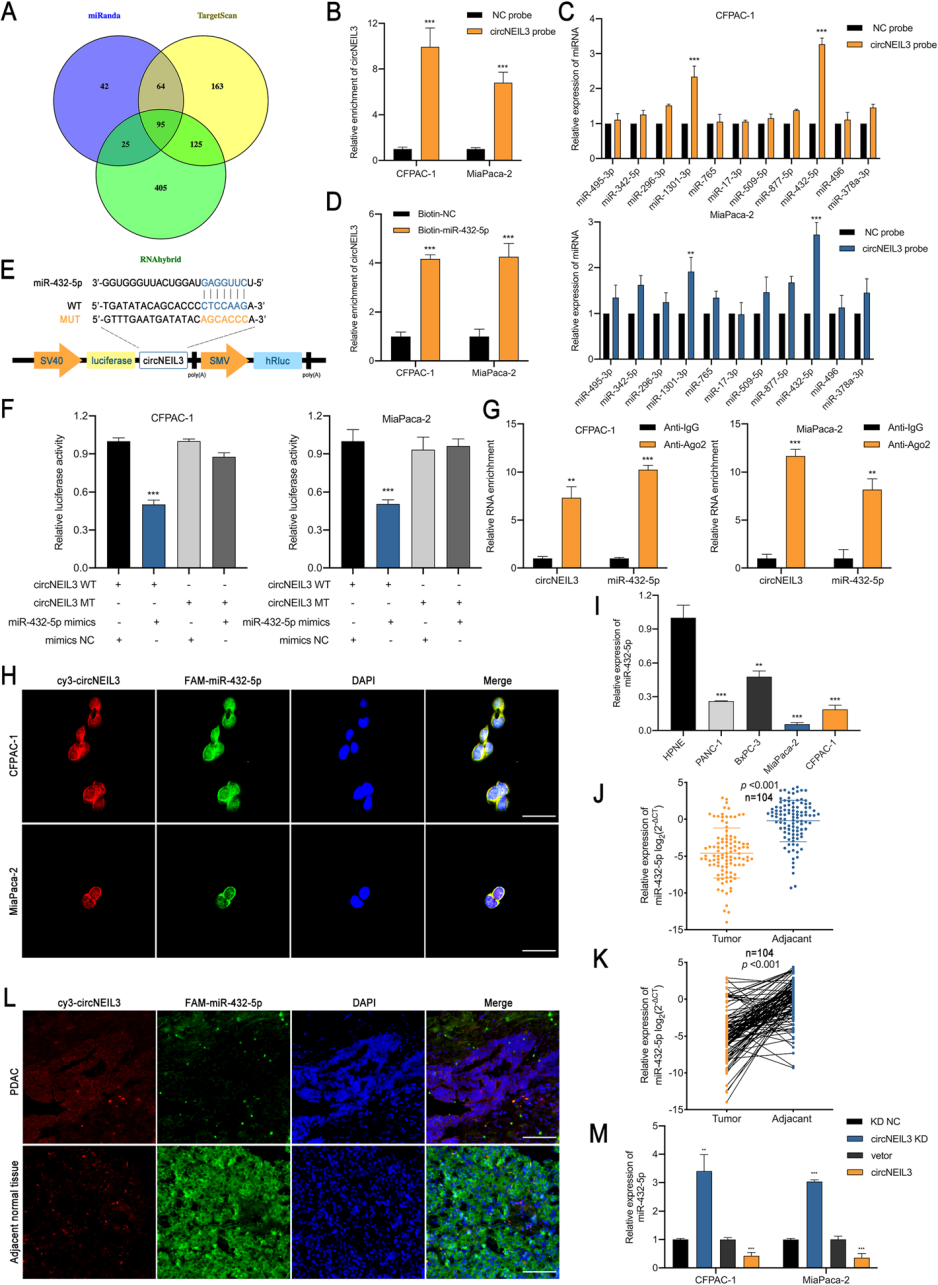
CircNEIL3 serves as a sponge for miR-432-5p. **a**. Venn diagram showing the overlap of the target miRNAs of circNEIL3 predicted by miRanda, TargetScan and RNAhybrid**. b**. The efficiency of the circNEIL3 probe in PDAC cells was validated using RT-qPCR after the RNA pull-down assay. A random sequence NC probe served as a negative control. **c.** The relative levels of 11 miRNA candidates in CFPAC-1 and MiaPaca-2 lysates were detected by RT-qPCR. **d**. Biotinylated miRNA pull-down in PDAC cells, and RT-qPCR results showing circNEIL3 expression levels. A random sequence NC probe served as a negative control. **e**. A schematic of the wild-type (WT) and mutant (MUT) circNEIL3 luciferase reporter vectors. **f**. The luciferase activities of the circNEIL3 luciferase reporter vector (WT or MUT) in CFPAC-1 and MiaPaca-2 cells transfected with miR-432-5p mimics or mimic NC. **g**. Anti-Ago2 RIP was performed using PDAC cells followed by RT-qPCR to detect circNEIL3 and miR-432-5p. **h**. The colocalization of circNIEL3 and miR-432-5p in PDAC cells was detected using a FISH assay. The circNIEL3 probe was labeled with Cy3 (red), miR-432-5p probes were labeled with FAM (green), and nuclei were stained with DAPI (blue). The samples were imaged at 1000× magnification. Scale bar = 10 μm. **i**. RT-qPCR analysis of the relative expression levels of miR-432-5p in pancreatic epithelial cells (HPNE) and PDAC cell lines. **j-k**. The relative expression of miR-432-5p was detected in 104 paired PDAC tissues and adjacent normal tissues by RT-qPCR. **l**. FISH results showing the colocalization of circNIEL3 and miR-432-5p in PDAC and adjacent normal tissues from patients. The samples were imaged at 400× magnification. Scale bar = 25 μm. **m**. The relative expression of miR-432-5p in cells was detected by RT-qPCR after transfection with indicated vectors. All data are presented as the means ± SD of three independent experiments. *p < 0.05, **p < 0.01, ***p < 0.001

CircRNAs have been reported to primarily function as miRNA sponges to regulate the expression of downstream genes [6]. Therefore, taking the sequence of circNEIL3 as a bait, we first performed a cross-analysis using three miRNA target prediction databases (miRanda, TargetScan and RNAhybrid) and identified 95 candidate miRNAs that may bind to circNEIL3 (Fig. 4a and Tab. S4) [22–24]. Based on the conjugation scores, we selected the top 11 miRNAs (miR-495-3p, miR-342-5p, miR-296-3p, miR-1301-3p, miR-765, miR-17-3p, miR-509-5p, miR-877-5p, miR-432-5p, miR-496, and miR-378a-3p) for further study. Next, we designed a biotin-labeled circNEIL3 probe to indirectly capture the potential binding miRNA in RNA pull-down assays. To test the specificity of the probe, RT-qPCR was used to assess the levels of circNEIL3 remaining after performing pull-down experiments compared to the random sequence NC probe. The results showed that circNEIL3 was mostly captured by the circNEIL3 probe in CFPAC-1 and MiaPaca-2 cells (Fig. 4b). Furthermore, miR-432-5p was the most highly enriched miRNA in the sponge complexes for both PDAC cell lines (Fig. 4c). Accordingly, biotin labelled miR-432-5p could directly pull down circNEIL3, ruling out the possibility of false positives due to indirect pull-down assays (Fig. 4d). To identify the specific binding region between circNEIL3 and miR-432-5p, a dual-luciferase reporter assay was performed. Based on the complementary base pairing prediction with the “seed” region of miR-432-5p, we mutated the predicted binding site of circNEIL3 and inserted the mutant sequence downstream of the luciferase reporter gene (Fig. 4e). After cotransfecting CFPAC-1 and MiaPaca-2 cells with miR-432-5p mimics and a circNEIL3-WT reporter gene, the observed luciferase activity was significantly reduced. However, miR-432-5p mimics or mimics NC showed no significant difference in luciferase activity when cotransfected with mutant reporter, indicating that circNEIL3 could directly and specifically bind to miR-432-5p at the “seed” region (Fig. 4f). In general, miRNAs function as RISC components, binding to Argonaute-2 (AGO2) [25]. Accordingly, an anti-AGO2 RNA immunoprecipitation (RIP) assay was performed, and the results showed that both circNEIL3 and miR-432-5p were pulled down by the anti-AGO2 antibody but not by IgG (Fig. 4g). FISH analysis showed that circNEIL3 and miR-432-5p colocalized in the cytoplasms of CFPAC-1 and MiaPaca-2 cells (Fig. 4h). Additionally, in contrast to circNEIL3, miR-432-5p was notably downregulated in both PDAC cell lines and tissues (Fig. 4i-k). Moreover, the FISH results showed that circNEIL3 was primarily and highly expressed in the adenocarcinoma portion of PDAC tissues, while miR-432-5p was mostly located in acinous cells in the adjacent normal tissue (Fig. 4l). Moreover, circNEIL3 in CFPAC-1 and MiaPaca-2 cells negatively regulated the expression of miR-432-5p after knocking down or overexpressing circNEIL3 (Fig. 4m). These data demonstrate that circNEIL3 acts as a sponge of miR-432-5p and suppresses its expression.

### MiR-432-5p reverses the oncogenic effects of circNEIL3 in PDAC cells

**Fig. 5:**
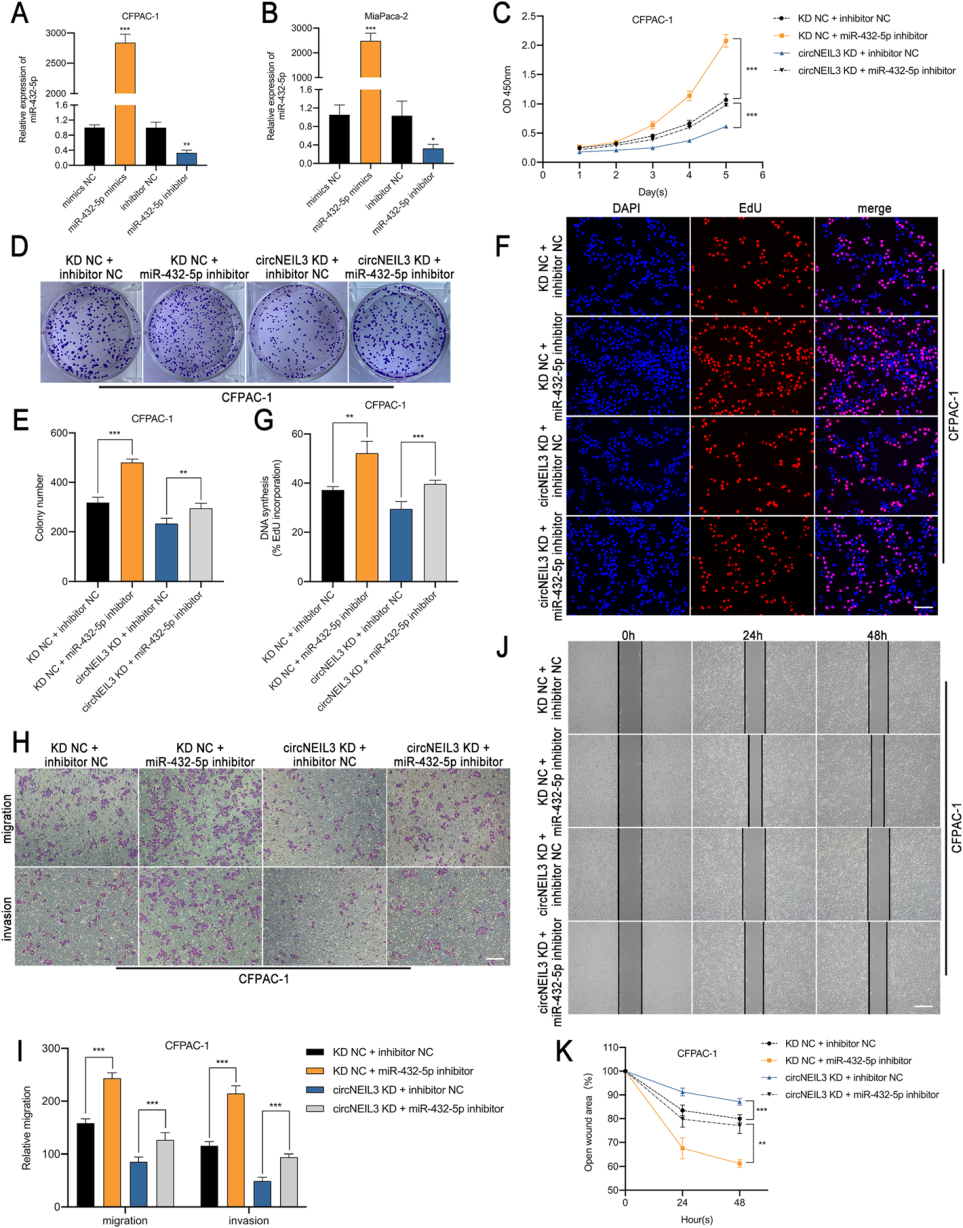
MiR-432-5p reverses the oncogenic effects of circNEIL3 in CFPAC-1 cells. **a-b**. The expression of miR-432-5p in CFPAC-1 and MiaPaca-2 cells transfected with the miR-432-5p mimics, inhibitor and corresponding NC was detected by RT-qPCR. **c-k**. CFPAC-1 cells were divided into four groups (circNEIL3 KD NC + miR-432-5p inhibitor NC, KD NC + miR-432-5p inhibitor, circNEIL3 KD + inhibitor NC and circNEIL3 KD + miR-432-5p inhibitor). The proliferation, migration and invasion ability of CFPAC-1 was analyzed through CCK-8, colony formation, EdU, transwell and wound healing assays. The EdU samples were imaged at 200× magnification. Scale bar = 50 μm. The transwell and wound healing images taken at 100× magnification. Scale bar = 100 μm. All data are presented as the means ± SD of three independent experiments. *p < 0.05, **p < 0.01, ***p < 0.001, ****p < 0.0001

To elucidate whether circNEIL3 functions by sponging miR-432-5p, rescue experiments were performed with cotransfection of miR-432-5p mimics or their inhibitor with LV-si-circNEIL3 or circNEIL3 plasmid. The efficiency of miR-432-5p mimics and inhibitor on its expression level in CFPAC-1 and MiaPaca-2 cells was verified by RT-qPCR (Fig. 5a-b). The results indicated that the miR-432-5p inhibitor significantly promoted the proliferation, migration and invasion of CFPAC-1 cells and reversed the suppressive effects on these processes induced by circNEIL3 downregulation in CCK-8, colony formation, EdU, Transwell and wound healing assays (Fig. 5c-k). Similar results were observed after transfection with miR-432-5p mimics and the circNEIL3 plasmid in MiaPaca-2 cells (Fig. S2a-i). Collectively, these data demonstrate that miR-432-5p exerts an anti-oncogenic effect on PDAC cells and serves a crucial function downstream of circNEIL3.

### ADAR1 is a downstream target of miR-432-5p and is indirectly regulated by circNEIL3

**Fig. 6:**
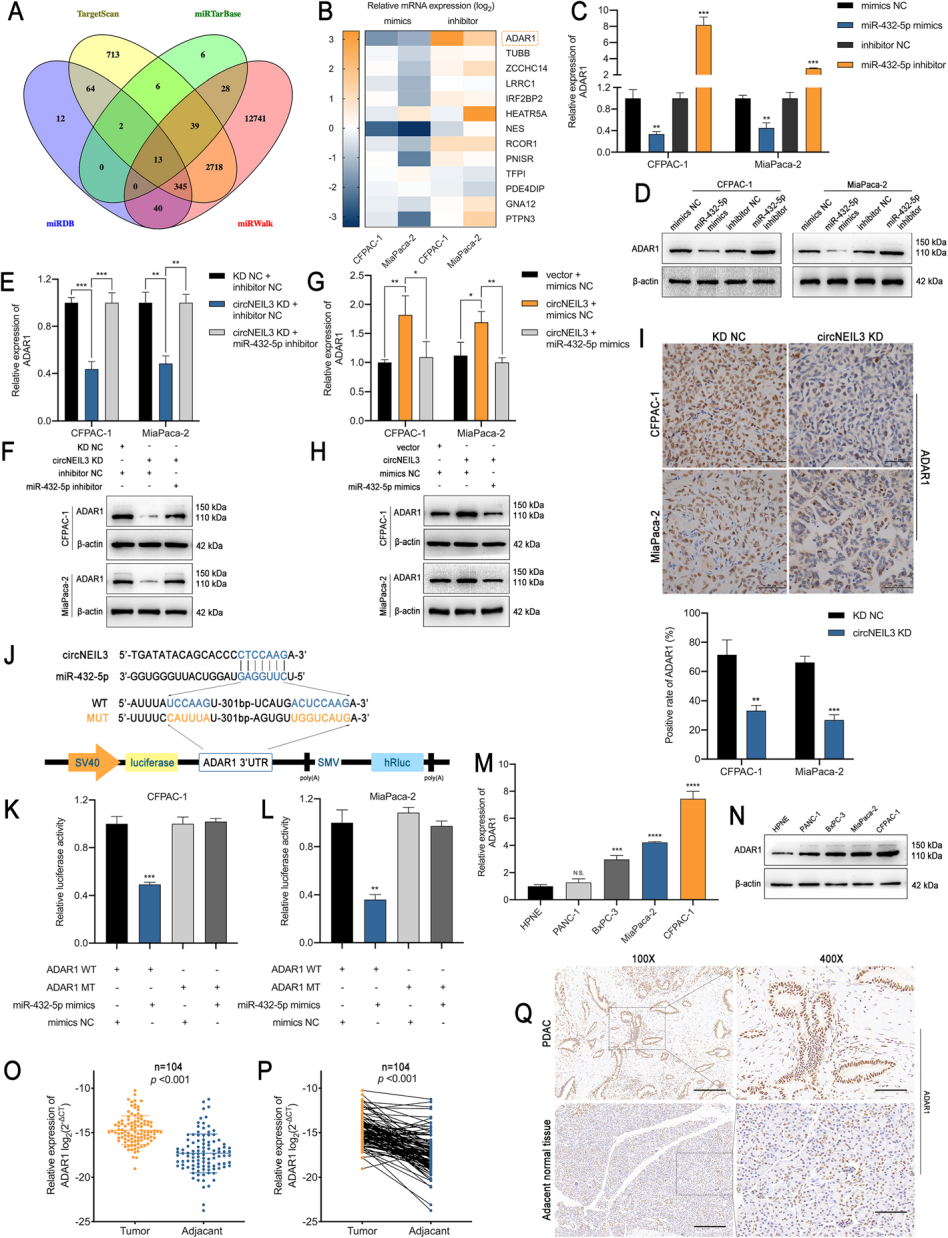
ADAR1 is a direct target of miR-432-5p and is indirectly regulated by circNEIL3 **a**. Venn diagram showing 13 genes that are putative miR-432-5p targets predicted by four algorithms (Targetscan, miRTarBase, miRDB and miRWalk). **b**. A heat map was used to visualize the expression of the predicted target genes in CFPAC-1 and MiaPaca-2 cells transfected with the miR-432-5p mimics, inhibitor and corresponding NC. **c-d**. ADAR1 expression in CFPAC-1 and MiaPaca-2 cells was analyzed by RT-qPCR and western blot analyses after transfection with the miR-432-5p mimics or inhibitor. **e-h**. ADAR1 expression was analyzed by RT-qPCR and western blot analyses. CFPAC-1 and MiaPaca-2 cells were transfected with the miR-432-5p mimic or cotransfected with the indicated circNEIL3 vectors. **i**. IHC staining of xenograft tumours. The protein levels of ADAR1 were analyzed based on IHC staining. The samples were imaged at 400× magnification. Scale bar = 50 μm. **j**. Schematic of the ADAR1 3’UTR wild-type (WT) and mutant (MUT) luciferase reporter vectors. **k-l**. The relative luciferase activities were analyzed in CFPAC-1 and MiaPaca-2 cells cotransfected with the miR-432-5p mimics or mimics NC and the ADAR1 3’UTR wild-type (WT) or mutant (MUT) luciferase reporter vectors. **m-n.** RT-qPCR and western blot analyses of the relative expression levels of ADAR1 in HPNE, PANC-1, BxPC-3, MiaPaca-2 and CFPAC-1. **o-p.** RT-qPCR analysis of ADAR1 expression in PDAC tissues (*n* = 104) paired with adjacent normal tissues (n = 104). **q.** IHC staining of ADAR1 of PDAC and adjacent normal tissues from patients. The samples were imaged at 100× and 400× magnification. Scale bar = 100 and 25 μm. All data are presented as the means ± SD of three independent experiments. n.s., no significance; *p < 0.05, **p < 0.01, ***p < 0.001

To further elucidate the underlying mechanism associated with the miR-432-5p-mediated regulation of PDAC, we performed another bioinformatic analysis using the online programs TargetScan, miRTarBase, miRDB and miRWalk [24, 26–28]. The data revealed 13 possible genes targeted by miR-432-5p (Fig. 6a). Since miRNAs typically interact with the 3′-UTR of target genes and downregulate their expression, we screened the changes in the expression of these genes in CFPAC-1 and MiaPaca-2 using miR-432-5p mimics or inhibitor with RT-qPCR (Fig. 6b). The RT-qPCR and western blot results identified ADAR1 as the gene most significantly and negatively regulated by miR-432-5p in both cell lines (Fig. 6c-d). Moreover, ADAR1 expression at both the mRNA and protein levels was positively correlated with the abundance of circNEIL3, which could be fully rescued by miR-432-5p mimics or inhibitor treatment (Fig. 6e-h). The IHC staining results for ADAR1 in the previously described subcutaneous tumour in the in-vivo model further confirmed the correlation between circNEIL3 and ADAR1 (Fig. 6i). Next, we constructed wild-type and mutant dual-luciferase reporter plasmids harbouring the wild-type or mutant ADAR1 3′-UTR (Fig. 6j), and the results showed that the transfection of cells with miR-432-5p mimics notably downregulated the luciferase activity in the plasmid with WT but not MUT 3′-UTR of ADAR1(Fig. 6k-l). Mechanistically, ADAR1 shared the same sequence with circNEIL3 that binds to the “seed” region of miR-432-5p, and similar to circNEIL3, ADAR1 was highly expressed in the PDAC cell lines, especially in CFPAC-1 and MiaPaca-2 cells (Fig. 6m-n). Clinically, the RT-qPCR and IHC results indicated that ADAR1 mRNA and protein expression was notably increased in PDAC tissues (Fig. 6o-q). Taken together, these results indicate that circNEIL3 antagonizes miR-432-5p-induced ADAR1 degradation and may function as an oncogene in PDAC.

### CircNEIL3 promotes PDAC progression via the miR-432-5p/ADAR1 axis

**Fig. 7:**
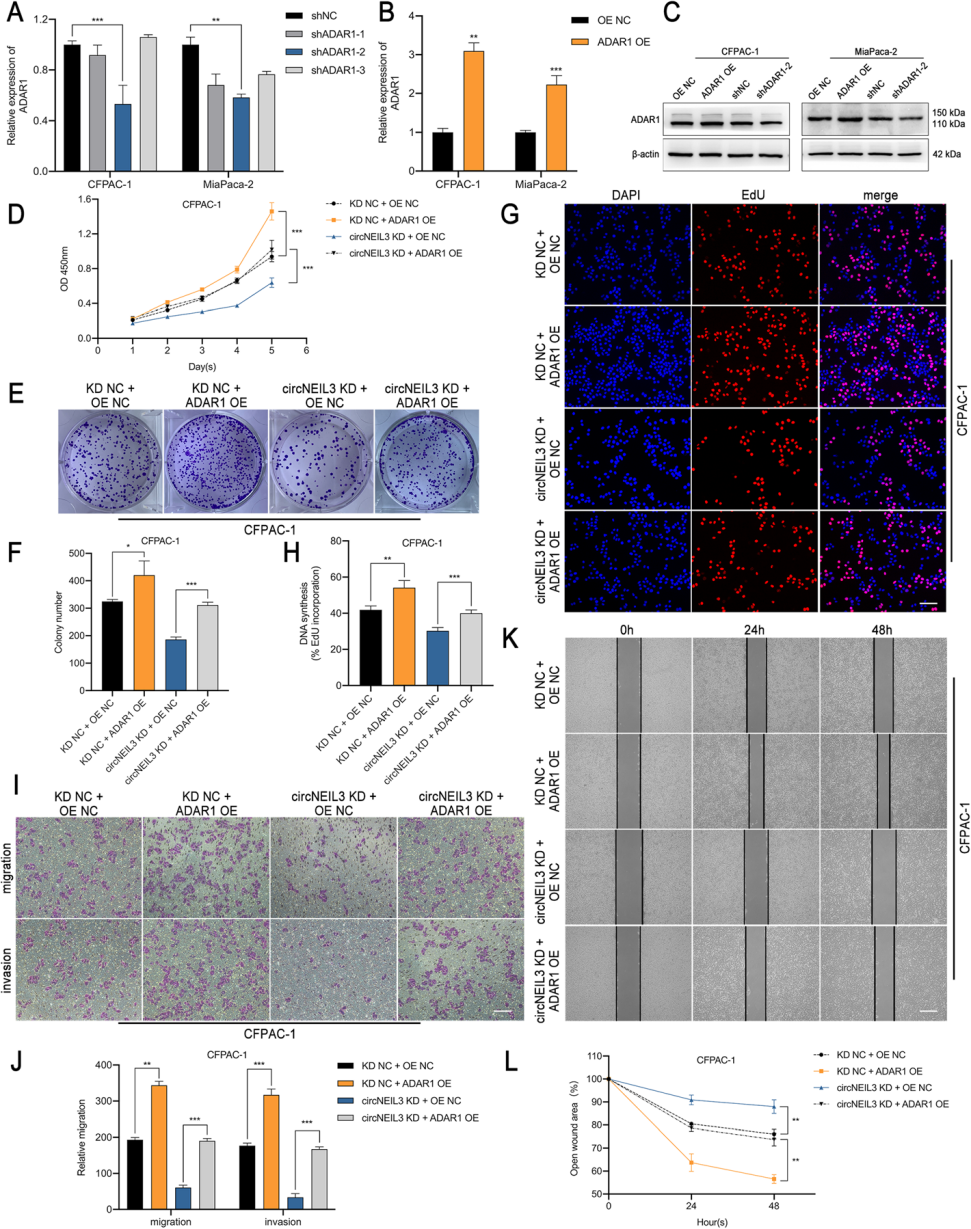
ADAR1 overexpression reverses the suppression induced by circNEIL3 downregulation **a-c**. The transfection efficiency of the ADAR1 shRNA and overexpression lentivirus in CFPAC-1 and MiaPaca-2 cells was verified by RT-qPCR and western blot analyses. **d-l**. CCK-8, colony formation, EdU, transwell and wound healing assay results demonstrated that transfection with the ADAR1 overexpression lentivirus increased the proliferation, migration, and invasion abilities of CFPAC-1 cells, which were reduced after cotransfection with the circNEIL3 KD lentivirus. The EdU samples were imaged at 200× magnification. Scale bar = 50 μm. The transwell and hound healing samples were imaged at 100× magnification. Scale bar = 100 μm. All data are presented as the means ± SD of three independent experiments. *p < 0.05, **p < 0.01, ***p < 0.001

As ADAR1 is a structural target that functions downstream of miR-432-5p and circNEIL3, we further evaluated whether circNEIL3 and ADAR1 are functionally associated. To regulate the ADAR1 RNA and protein levels, three short hairpin RNAs and an overexpression lentivirus were constructed and transfected into CFPAC-1 and MiaPaca-2 cells (Fig. 7a-c). Subsequently, with CCK-8, colony formation, EdU, Transwell and wound healing assays, ADAR1 overexpression was demonstrated to promote the proliferation, migration and invasion of CFPAC-1 cells (Fig. 7d-l). In contrast, ADAR1 knockdown had the opposite effects in MiaPaca-2 cells (Fig. S3a-i). Further, we observed that the inhibition of malignant biological behaviour after disrupting circNEIL3 expression could be largely blocked by ADAR1 overexpression (Fig. 7d-l), while the increased proliferation, migration and invasion capabilities of PADC cells caused by circNEIL3 overexpression could be retarded by ADAR1 knockdown (Fig. S3a-i). Collectively, our data suggest that the promoting role of circNEIL3 in maintaining the malignant phenotype of PDAC was largely dependent on the miR-432-5p/ADAR1 axis.

### ADAR1 enhances GLI1 RNA editing and functions via the cell cycle and EMT pathway

**Fig. 8:**
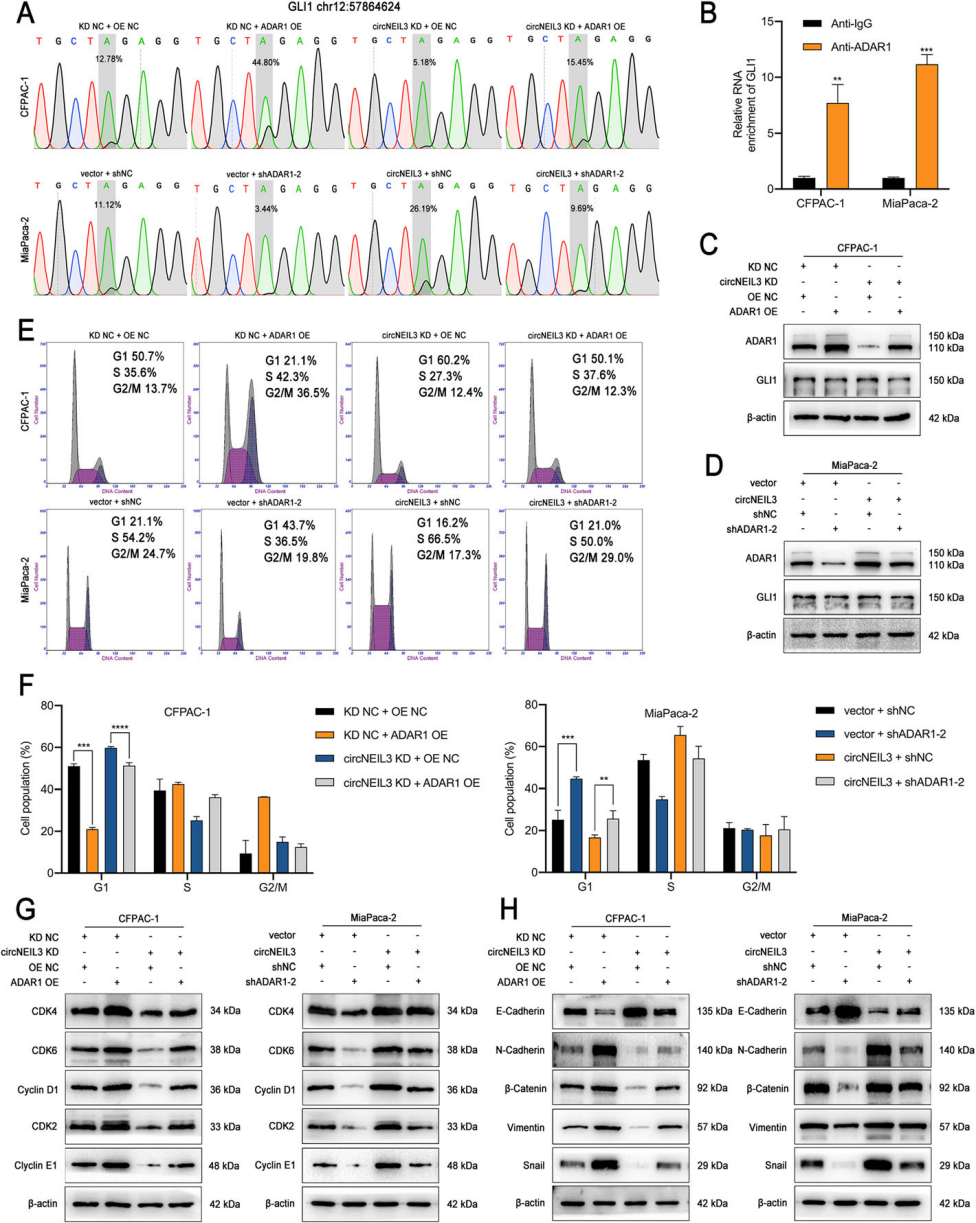
ADAR1 enhances GLI1 RNA editing and functions via the cell cycle and EMT pathway **a.** Representative Sanger sequencing chromatograms for GLI1 in the indicated cells. The gray box highlights the double A/G peak, labeled with the percentage of edited transcripts assessed as the edited allele burden (G/(G + A) %). **b.** RIP analyses for GLI1 in CFPAC-1 and MiaPaca-2 cells using an ADAR1 antibody. **c-d**. Western blot results revealed the expression of ADAR1 and GLI1 in cells transfected with the indicated shRNA vector. **e-f**. The cell cycle progression was analyzed by flow cytometry after cells were transfected with the indicated plasmids. **g**. The relative expression of cyclin D1 and downstream cell cycle-related molecules at the protein level in cells transfected with the indicated vectors and shRNAs was determined by western blot analysis. **h**. The expression of EMT protein markers in the indicated cells was detected by western blot analysis. All data are presented as the means ± SD of three independent experiments. *p < 0.05, **p < 0.01, ***p < 0.001

As ADAR1 could stimulate PDAC progression, and recent reports on whole genome screening for instances of RNA editing have shown that many mRNAs, especially GLI1, are edited in multiple malignancies [18], we further examined whether ADAR1 induces RNA editing of GLI1 in PDAC cells. To this end, Sanger sequencing was used to detect the A-to-I editing of GLI1 in CFPAC-1 and MiaPaca-2 cells, which was subsequently translated to substituted amino acids (A-to-G). Consistent with other studies, we observed that GLI1 mRNA is edited by the A-to-I conversion in exon 12, specifically at nucleotide position chr12:57864624 [29]. GLI1 transcripts were exhibited significant hyperediting after ADAR1 overexpression, while ADAR1 knockdown had the opposite effect. Moreover, increased circNEIL3 levels were positively associated with the rate of GLI1 mRNA editing and could be abrogated by cotransfection with an ADAR1 expression construct (Fig. 8a and Fig. S4a). Subsequently, we performed RNA immunoprecipitation (RIP) experiments using an anti-ADAR1 antibody with CFPAC-1 and MiaPaca-2 cells, and the results showed that endogenous ADAR1 directly bound to GLI1 mRNA (Fig. 8b). GLI1 transcript editing was previously reported to lead to an R701G (arginine to glycine) amino acid change [29]. Our western blot results showed that ADAR1 did not alter the GLI1 protein levels, indicating that RNA editing of the GLI1 transcript may alter the secondary structure of the GLI1 protein (Fig. 8c-d and Fig. S4b-c). Indeed, the target site for RNA editing is located within a domain in the C-terminus of GLI1 that binds to the negative regulator of HH signalling, suppressor of fused (SUFU) [29]. Furthermore, the edited GLI1 protein was previously reported to have a lower susceptibility to inhibition by SUFU, resulting in it having an increased ability to activate most transcriptional targets, including cyclin D1, cyclin E1, and Snail [30, 31]. Thus, a cell cycle analysis was performed, with the results showing that knockdown of ADAR1 or circNEIL3 led to higher percentages of CFPAC-1 and MiaPaca-2 cells in G0-G1 phase, while their overexpression had the opposite effect (Fig. 8e-f and Fig. S4d-e). Moreover, western blot analysis results demonstrated that ADAR1 upregulation enhanced the protein levels of the cyclin D1/CDK4/CDK6 complex, and the downstream targets cyclin E1 and CDK2, while ADAR1 knockdown decreased the levels of these proteins. These results could also be obtained by overexpressing or silencing circNEIL3 and could be reversed by ADAR1 knockdown or overexpression, respectively (Fig. 8g and Fig. S4f). In agreement with the observed ability of circNEIL3 to promote cellular proliferation in vitro and in vivo, these results indicated that circNEIL3 downregulation prevented PDAC cells from shifting from G1 to S phase by functioning as a ceRNA for miR-432-5p to regulate the ADAR1/GLI1/Cyclin D1/CDKs axis. Furthermore, as crucial downstream targets of GLI1, EMT-related proteins were detected by western blot analysis. The results showed that PDAC with depletion of ADAR1 and circNEIL3 had higher levels of N-cadherin and lower expression of E-cadherin, β-Catenin, vimentin and Snail than the control group (Fig. 8h and Fig. S4g). Similar results were observed for the overexpression and rescue groups, suggesting that circNEIL3 induces the transition of PDAC cells from an epithelial phenotype to mesenchymal phenotype by activating ADAR1/GLI1/Snail signalling.

### An Alu-dependent circNEIL3/miR-432-5p/ADAR1 feedback loop is correlated with PDAC prognosis

**Fig. 9:**
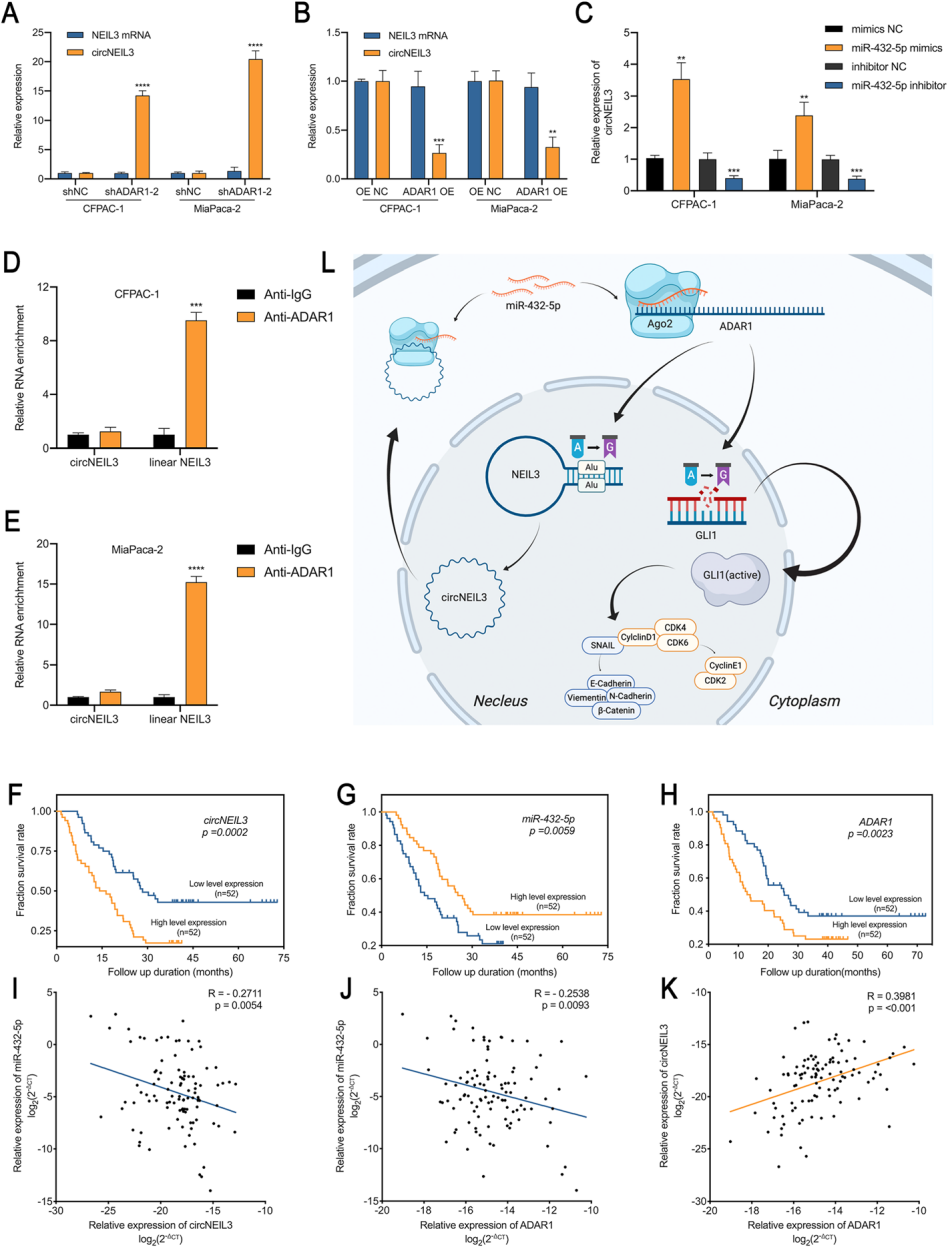
An Alu-dependent feedback loop involving circNEIL3/miR-432-5p/ADAR1 is correlated with the prognosis of PDAC patients. **a-b**. The expression levels of circNEIL3 and NEIL3 mRNA in CFPAC-1 and MiaPaca-2 cells transfected with indicated vectors or shRNA were determined by RT-qPCR. **c**. The level of circNEIL3 expression was analyzed by RT-qPCR. CFPAC-1 and MiaPaca-2 cells were transfected with the miR-432-5p mimics or inhibitor. **d-e**. RIP assays using an ADAR1 antibody were performed to capture circNEIL3 or NEIL3 mRNA. **f-h**. Kaplan-Meier survival curves showed the OS of PDAC patients with low vs. high circNEIL3, miR-432-5p or ADAR1 expression. The cutoff value was the median expression of these genes. **i-k**. Correlation analysis between circNEIL3, miR-432-5p and ADAR1 expression analyzed by RT-qPCR in PDAC tissues (*n* = 104). **l**. Schematic diagram illustrating the mechanism by which circNEIL3 promotes PDAC proliferation and metastasis through the circNEIL3/miR-432-5p/ADAR1/GLI1/cell cycle & EMT axis and is regulated by ADAR1 through a negative feedback loop. All data are presented as the means ± SD of three independent experiments. *p < 0.05, **p < 0.01, ***p < 0.001

Alu elements, which are often present in the flanking introns of circularized exons, are required for circRNA formation [6]. ADAR1 has been reported to recognize inverted Alu repeats and perform A-to-I editing to unwind the dsRNA structure and prevent splice sites from being close enough to back splice [32]. In NEIL3, we identified two Alu elements besides exons 8–9 in the introns 7 and 9, named AluY (position: chr4:178273703–178,273,999) and FLAM_C (position: chr4:178282936–178,283,074). The sequence of these two Alu elements are listed in Tab. S5. Therefore, we hypothesized that miR-432-5p may transcriptionally regulate circNEIL3 expression through ADAR1 in an Alu-dependent manner. To test our hypothesis, RT-qPCR and RIP assays were performed, and the results indicated that ADAR1-depleted cells showed greatly increased circNEIL3 levels, while linear NEIL3 were only slightly affected (Fig. 9a). Furthermore, circNEIL3 production was severely blocked after ADAR1 overexpression (Fig. 9b). As expected, we also observed that miR-432-5p overexpression and silencing in CFPAC-1 or MiaPaca-2 cells decreased or increased the circNEIL3 levels, respectively (Fig. 9c). Moreover, ADAR1 specifically bound to linear NEIL3 rather than to circNEIL3, indicating that ADAR1 participates in the formation of circNEIL3 (Fig. 9d-e).

Given the discovery of the circNEIL3/miR-432-5p/ADAR1 loop in PDAC cells and that the positive relationship between miR-432-5p and circNEIL3 conflicted with the negative correlation demonstrated above, we next assessed the clinical significance of circNEIL3, miR-432-5p and ADAR1 in a cohort of 104 PDAC patients to crystallize the principal line of the story. Patients were divided into two groups based on their median expression. Importantly, a Kaplan-Meier analysis revealed that higher circNEIL3 expression, lower miR-432-5p expression and higher ADAR1 expression were associated with the poorer overall survival (OS) in104 PDAC patients (Fig. 9f-h). The relationship between circNEIL3, miR-432-5p and ADAR1 expression and the clinical characteristics of the PDAC patients are listed in Table 1. Tumour size, microscopic vascular and nerve invasion and postoperative liver metastasis were statistically significantly associated with at least one of these three biomarkers. Further univariate and multivariate Cox regression analysis showed that the circNEIL3 and ADAR1 expression levels were independent prognostic factors for PDAC patients, as were tumour size and TNM stage (Table 2). Furthermore, Pearson correlation analysis showed that circNEIL3 and ADAR1 expression were negatively correlated with miR-432-5p expression levels, while circNEIL3 was positively correlated with ADAR1 (Fig. 9i-k). Taken together, these results indicate the existence of a circNEIL3/miR-432-5p/ADAR1 axis in PDAC with clinical significance, while miR-432-5p positively regulates circNEIL3 in an Alu-dependent manner via ADAR1 as a part of hemostasis of endogenous circNEIL3.

**Table 1:** Association of circNEIL3, miR-432-5p and ADAR1 expression with clinicopathological features of PDAC (*n* = 104)

Clinicopathological features	circNEIL3 expression		*P* value	miR-432-5p expression		*P* value	ADAR1 expression		*P* value
	High	Low		High	Low		High	Low	
All cases	52	52		52	52		52	52	
Gender			0.676			0.012*			0.676
Male	36	34		29	41		34	36	
Female	16	18		23	11		18	16	
Age (year)			0.527			0.527			0.527
< 60	18	15		18	15		15	18	
≥ 60	34	37		34	37		37	34	
Serum CA199 (U/ml)			0.129			0.319			0.292
< 39	7	10		11	6		7	10	
≥ 39	31	36		33	34		32	35	
≥ 1000	14	6		8	12		13	7	
Diameter (cm)			0.098			0.033*			0.033*
≤ 4	37	44		45	36		36	45	
> 4	15	8		7	16		16	7	
Location			0.094			0.403			0.676
Head	39	31		37	33		36	34	
Body and tail	13	21		15	19		16	18	
Histological grade			0.543			0.311			0.203
I/ I-II	34	31		35	30		32	33	
II/ II-III/ III	18	21		17	22		20	19	
Microscopic vascular invasion			0.02*			0.527			0.833
Present	41	30		34	37		35	36	
Absent	11	22		18	15		17	16	
Microscopic nerve invasion			0.014*			0.066			0.22
Present	50	42		49	43		48	44	
Absent	2	10		3	9		4	8	
T stage			0.168			0.844			0.554
T1/T2	25	32		29	28		27	30	
T3/T4	27	20		23	24		25	22	
N stage			0.325			0.554			0.076
N0	21	26		22	25		28	19	
N1/N2	31	26		30	27		24	33	
M stage			0.475			0.475			0.475
M0	50	52		52	50		50	52	
M1	2	0		0	2		2	0	
Clinical stage			0.146			0.534			0.299
I-IIa	14	21		16	19		20	15	
IIb-IV	38	31		36	33		32	37	

All data are presented as the mean ± SD. *p < 0.05, **p < 0.01, ***p < 0.001

**Table 2:** Univariate and multivariate analyses of prognostic factors in PDAC patients (*n* = 104)

Variable	Univariate analysis				Multivariate analysis		
	Cases	Events	Median survival (months)	*P* value	HR	95%CI	*P* value
Gender, male/ female	70/ 34	48/ 24	22.1/ 19	0.8936			
Age, < 60/ ≥60	33/ 71	19/ 53	27.4/ 18.5	0.0836			
Serum CA199 (U/ml)							
< 39, ≥39	17/ 67	10/ 48	20.0/ 12.7	0.348			
≥ 39, ≥1000	67/ 20	48/ 14	12.7/ 11.0	0.543			
< 39, ≥1000	17/ 20	10/ 14	20.0/ 11.0	0.078			
Location, head/ body or tail	70/ 34	49/ 23	20/ 16.9	0.4537			
Diameter, ≤4/ > 4	81/ 23	51/ 21	25.3/ 9.1	< 0.0001****	1.594	1.224–2.075	0.001**
Histological grade, I, I-II/ II, II-III, III	65/ 39	44/ 28	21.9/ 19.2	0.7374			
Microscopic vascular invasion, absent/ present	33/ 71	19/ 53	30.3/ 18.2	0.0146*			0.537
Microscopic nerve invasion, absent/ present	12/ 92	8/ 64	22.4/ 19.8	0.9094			
T stage, T1, 2/ T3, 4	57/ 47	34/ 38	27.6/ 12.9	0.0004***			0.204
N stage, N0/ N1,2	47/ 57	30/ 42	25.3/ 18.2	0.07			
M stage, M0/ M1	102/ 2	70/ 2	20/ 13.6	0.2423			
Clinical stage, I-IIa/ IIb-IV	35/ 69	19/ 53	30.3/ 18.2	0.0051**	2.127	1.235–3.665	0.007**
circNEIL3 expression, low/ high	52/ 52	29/ 43	28.4/ 15.3	0.0002***	1.995	1.225–3.248	0.006**
miR-432-5p expression, low/ high	52/ 52	40/ 32	14.8/ 26.1	0.0059**			0.137
ADAR1 expression, low/ high	52/ 52	32/ 40	25.4/ 13.4	0.0023**	1.765	1.079–2.889	0.024*

*p < 0.05, **p < 0.01, ***p < 0.001, ****p < 0.0001

## Discussion

Emerging evidence indicates that circRNAs, as novel noncoding RNAs, play important roles in multiple cancers and may be involved in the pleiotropic modulation of cellular functions [33]. For instance, many circRNAs function as miRNA sponges to regulate gene expression, bind to specific proteins to influence their functions or encode polypeptides [5].

In the present study, we profiled circRNA expression in 3 pairs of PDAC tumour tissues and adjacent normal tissues by RNA sequencing, resulting in the identification of 203 differentially expressed circRNAs, including 79 upregulated and 124 downregulated circRNAs. For the first time, we identified circNEIL3 as a significantly upregulated circRNA in pancreatic ductal adenocarcinoma tissues and cell lines. Loss- and gain-of-function experimental results suggested that circNEIL3 promotes the proliferation, migration and invasion of PDAC cells in vitro and in vivo, indicating an oncogenic role of circNEIL3 in PDAC and its potential value as a biomarker to predict the prognosis of PDAC patients.

Accumulating evidence has revealed that circRNAs primarily function as miRNA sponges. Zhang. et al. reported that cicFGFR1 acts as a miR-381-3p sponge to promote the progression and anti-PD-1 resistance of NSCLC [34]. Wang et al. showed the sponging activity of circLMTK2 towards miR-150-5p, which was demonstrated to be essential for the proliferation and metastasis of gastric cancer [35]. In addition, Xie et al. observed that circBCRC-3 can suppress bladder cancer proliferation through miR-182-5p suppression [36]. In the present study, through cross-analysis of three miRNA target prediction databases (miRanda, TargetScan and RNAhybrid), we observed that circNEIL3 may accommodate multiple potential binding sites for 11 miRNAs. Subsequently, after performing pull-down assays with a biotin-labelled circNEIL3 probe, we observed that miR-432-5p was the most highly enriched miRNA in the sponge complexes with circNEIL3. To validate the binding between circNEIL3 and miR-432-5p, we performed dual-luciferase, FISH, and anti-AGO2 RNA immunoprecipitation (RIP) assays. The results indicated that, as an RISC complex, circNEIL3 could directly bind to the seed region of miR-432-5p in the cytoplasm of PDAC cells. Moreover, to elucidate the biological function of the complex, a rescue experiment was performed, and the results showed that the circNEIL3 knockdown-induced suppression of tumour proliferation and migration was reversed by treatment with a miR-432-5p inhibitor. Our results provide evidence to support the conjecture that circNEIL3 functions as a miRNA sponge that is important for the progression and metastasis of PDAC.

Several studies have revealed the antitumour activity of miR-432-5p, with Zhang et al. reporting that miR-432-5p can mediate cisplatin resistance in NSCLC through its ceRNA function towards the long noncoding RNA MSTRG.51053.2 [37]. In addition, Xu et al. observed that miR-432-5p can could suppress pancreatic carcinoma progression by downregulating PPME1 [38]. To further elucidate the underlying mechanism of miR-432-5p, we conducted bioinformatic, RT-qPCR and western blot analyses, with the results identifying ADAR1 as the most likely target of miR-432-5p. Dual-luciferase assay results demonstrated that the ADAR1 3’UTR shares the same binding site as circNEIL3 in the miR-432-5p ‘seed’ region. Moreover, similar to circNEIL3, ADAR1 is also upregulated in PDAC cells and tissues, leading us to wonder whether circNEIL3 promotes PDAC progression through the circNEIL3/miR-432-5p/ADAR1 axis. After constructing ADAR1 shRNA and overexpression lentiviral constructs, we observed that ADAR1 could remarkably promote the proliferation, migration and invasion of PDAC cell lines. Notably, the results of rescue experiments showed that ADAR1 overexpression could mediate the inhibitory effect of circNEIL3. Taken together, our results reveal that circNEIL3 acts as an oncogene in PDAC through the circNEIL3/miR-432-5p/ADAR1 axis.

Widely reported as an oncogene in cancer, ADAR1 is a key member of the ADAR enzyme family that facilitates adenosine-to-inosine (A-to-I) editing in double-stranded RNA (dsRNA) [14, 39, 40]. ADAR1-driven activation of AZIN1 RNA editing has been reported to promote the invasive potential of cancer-associated fibroblasts in colorectal cancer (CRC) [41]. ADAR1 was shown to enhance the Alu-dependent editing activity and transcription of GLI1, and the Sanger sequencing results demonstrated that the GLI1 transcript was hyperedited upon ADAR1 overexpression in PDAC cells [18], which involved the A-to-I conversion at nucleotide position chr12:57864624. The binding relationship between ADAR1 and GLI1 mRNA was demonstrated through RIP experiments using an anti-ADAR1 antibody. In addition, the GLI1 level was unaltered by ADAR1 expression, demonstrating that ADAR1 enhances GLI1 editing in PDAC cells. Furthermore, GLI1 editing was shown to promote the transcription of target genes, including cyclin D1, cyclin E and Snail [20, 42]. Subsequently, flow cytometry assay results showed that either circNEIL3 or ADAR1 knockdown led to more PDAC cells in G0-G1 phase, while the protein expression of cyclin D1, cyclin E1 and the cyclin-dependent kinases CDK2, CDK4 and CDK6 decreased. Moreover, rescue experiments further showed that circNEIL3 has a bidirectional effect on the cell cycle. Taken together, these results demonstrated the importance of the circNEIL3/miR-432-5p/ADAR1/GLI1/cyclin D1/CDK axis in PDAC progression. Given that circNEIL3 could promote the migration and invasion of PDAC cells via ADAR1 and that GLI1 has been reported to be associated with EMT signalling, we wondered whether the effect of ADAR1 on cell migration and invasion is dependent upon the EMT pathway through GLI1 [42]. As expected, the western blot results showed that the levels of the EMT-related proteins Snail, E-cadherin, β-Catenin and vimentin were significantly lower upon ADAR1 depletion than in the control group, indicating that circNEIL3 promotes PDAC cell migration by promoting EMT via the ADAR1/GLI1/Snail pathway.

Intriguingly, after searching the existing literature and performing further RIP and RT-qPCR assays, we showed that miR-432-5p can transcriptionally regulate the circularization of circNEIL3 in an Alu-dependent manner via ADAR1. Taken together, the results of the present study demonstrate the existence of a circNEIL3/miR-432-5p/ADAR1/GLI1 axis in PDAC cells. Finally, we demonstrated the existence of a feedback loop in this signalling axis in pancreatic ductal adenocarcinoma. Moreover, the clinical statistics showed powerful relationships among the three factors, demonstrating their significant value and central role in PDAC occurrence and development. The negative regulation of circNEIL3 by ADAR1 functions more as a part of the complex regulatory network of oncogenesis. However, the specific binding site between ADAR1 and circNEIL3 needs to be further illustrated to explore a brand-new idea for circRNA biogenesis regulation.

## Conclusion

In summary, for the first time, we identified the novel circRNA, circNEIL3, that may be an important marker in PDAC. In vivo assay results showed that circNEIL3 promotes tumour progression and metastasis, and correlation analysis of clinical PDAC samples showed that high circNEIL3 levels were associated with poor prognosis or TNM stage. Further univariate and multivariate analyses suggested that high circNEIL3 expression is an independent risk factor for PDAC survival. In addition, the circNEIL3/miR-432-5p/ADAR1 axis was shown to form a regulatory loop that regulates the proliferation and metastasis of PDAC via the downstream GLI/cell cycle and EMT pathway, providing a prognostic marker and therapeutic target for PDAC (Fig. 9l).

## Supplementary Information

**Additional file 1: Table S1.** The original sequencing results of all differentially expressed circRNAs in three pairs of PDAC and adjacent normal tissues.

**Additional file 2: Table S2.** Primers, probes and siRNAs used in the present study.

**Additional file 3: Table S3.** Antibodies used in the present study.

**Additional file 4: Table S4.** 95 candidate miRNAs binding to circNEIL3 predicted by cross-analyzing three miRNA target prediction databases.

**Additional file 5: Table S5.** The sequence of the two Alu elements besides circNIEL3 in NEIL3.

**Additional file 6: Figure S1.** CircNEIL3 promotes the proliferation, migration and invasion of PDAC cells in vitro. **a** RT-qPCR analysis of circNEIL3 and NEIL3 mRNA expression in CFPAC-1 and MiaPaca-2 cells transfected with si-circNEIL3. **b-c.** Colony formation assays were performed to evaluate cell proliferation. **d-e.** EdU assays of PDAC cells were performed to evaluate cell proliferation. The samples were imaged at 200× magnification. Scale bar = 50 μm. **f-g.** Transwell assays were performed to assess the migration and invasion abilities of PDAC cells. The samples were imaged at 100× magnification. Scale bar = 100 μm. **h-i.** Cell migration was determined using a wound healing assay. The samples were imaged at 100× magnification. Scale bar = 100 μm. All data are presented as the means ± SD of three independent experiments. *p < 0.05, **p < 0.01, ***p < 0.001, ****p < 0.0001.

**Additional file 7: Figure S2.** MiR-432-5p reverses the oncogenic effects of circNEIL3 in MiaPaca-2 cells. **a-i.** MiaPaca-2 cells were divided into four groups (circNEIL3 vector + miR-432-5p mimics NC, vector + miR-432-5p mimics, circNEIL3 + mimics NC and circNEIL3 + miR-432-5p mimics). The proliferation, migration and invasion abilities of MiaPaca-2 cells was analyzed through CCK-8, colony formation, EdU, transwell and wound healing assays. The EdU samples were imaged at 200× magnification. Scale bar = 50 μm. The transwell and wound healing samples were imaged at 100× magnification. Scale bar = 100 μm. All data are presented as the means ± SD of three independent experiments. *p < 0.05, **p < 0.01, ***p < 0.001, ****p < 0.0001.

**Additional file 8: Figure S3** ADAR1 downregulation reverses the oncogenic phenotype induced by circNEIL3 overexpression. **a-i.** CCK-8, colony formation, EdU, transwell and wound healing assay results showed that transfection with the ADAR1 shRNA inhibited the proliferation, migration, and invasion abilities of MiaPaca-2 cells, which was reversed after cotransfection with the circNEIL3 plasmid. The EdU samples were imaged at 200× magnification. Scale bar = 50 μm. The transwell and hound healing samples were imaged at 100× magnification. Scale bar = 100 μm. All data are presented as the means ± SD of three independent experiments. *p < 0.05, **p < 0.01, ***p < 0.001, ****p < 0.0001.

**Additional file 9: Figure S4.** ADAR1 enhances GLI1 RNA editing and functions via the cell cycle and EMT pathway. **a.** Representative Sanger sequencing chromatograms for GLI1 in the indicated cells. The gray box highlights the double A/G peak, labeled with the percentage of edited transcripts assessed as the edited allele burden (G/(G + A) %). **b-c**. Western blot results revealed ADAR1 and GLI1 expression in cells transfected with the indicated shRNA vector. **e-f.** The cell cycle progression was analyzed by flow cytometry after transfected with indicated plasmids. **g.** Western blot analysis of the relative protein levels of cyclin D1 and downstream cell cycle-related molecules in cells transfected with the indicated vectors and shRNAs. **h.** EMT protein markers in the indicated cells were detected by western blot analysis. All data are presented as the means ± SD of three independent experiments. *p < 0.05, **p < 0.01, ***p < 0.001, ****p < 0.0001.

## Data Availability

All data generated or analyzed during this study are included in this published article.

## Abbreviations

circRNA: Circular RNA
PDAC: Pancreatic ductal adenocarcinoma
SRPBM: Spliced reads per billion mapping
qRT-PCR: Quantitative reverse transcription polymerase chain reaction
RIP: RNA immunoprecipitation
FISH: Fluorescent in situ hybridization
ADAR1: Adenosine deaminases acting on RNA 1
GLI1: Glioma-associated oncogene 1
EMT: Epithelial-to-mesenchymal transition
miRNA: micro RNA
RBPs: RNA binding proteins
3’UTR: 3′-untranslated regions
RISC: RNA-induced silencing complex
A-to-I: Adenosine to inosine
dsRNA: Double-stranded RNA
NEIL3: Nei like DNA glycosylase 3
DAPI: 4,6-diamidino-2-phenylindole
CCK-8: Cell count kit-8
EdU: 5-ethynyl-20-deoxyuridine
Ago2: Argonaute-2
shRNA: Short hairpin RNA
IHC: Immunohistochemistry
DMEM: Dulbecco’s modified Eagle’s medium
FBS: Fetal calf serum
