# Correction: CircNEIL3 regulatory loop promotes pancreatic ductal adenocarcinoma progression via miRNA sponging and A-to-IRNA-editing

**DOI:** 10.1186/s12943-022-01636-3

**Published:** 2022-08-19

**Authors:** Peng Shen, Taoyue Yang, Qun Chen, Hao Yuan, Pengfei Wu, Baobao Cai, Lingdong Meng, Xumin Huang, Jiaye Liu, Yihan Zhang, Weikang Hu, Yi Miao, Zipeng Lu, Kuirong Jiang

**Affiliations:** 1 Pancreas Center, the First Affiliated Hospital of Nanjing Medical University, Nanjing, China; 2 Pancreas Institute, Nanjing Medical University, Nanjing, China; 3 Nanjing Medical University, Nanjing, China

**Correction: Mol Cancer 20, 51 (2021)**

**https://doi.org/10.1186/s12943-021-01333-7**

In our BMC Research publication in Molecular Cancer entitled ‘CircNEIL3 regulatory loop promotes pancreatic ductal adenocarcinoma progression via miRNA sponging and A-to-I RNA-editing ’[1], we misplaced two images in Fig. 2C and Fig.S3D. These errors inadvertently happened during the stage of our figure assembly with photoshop software. The corrected version of Fig. 2 and Fig.S3 has been provided.

**Fig 2:**
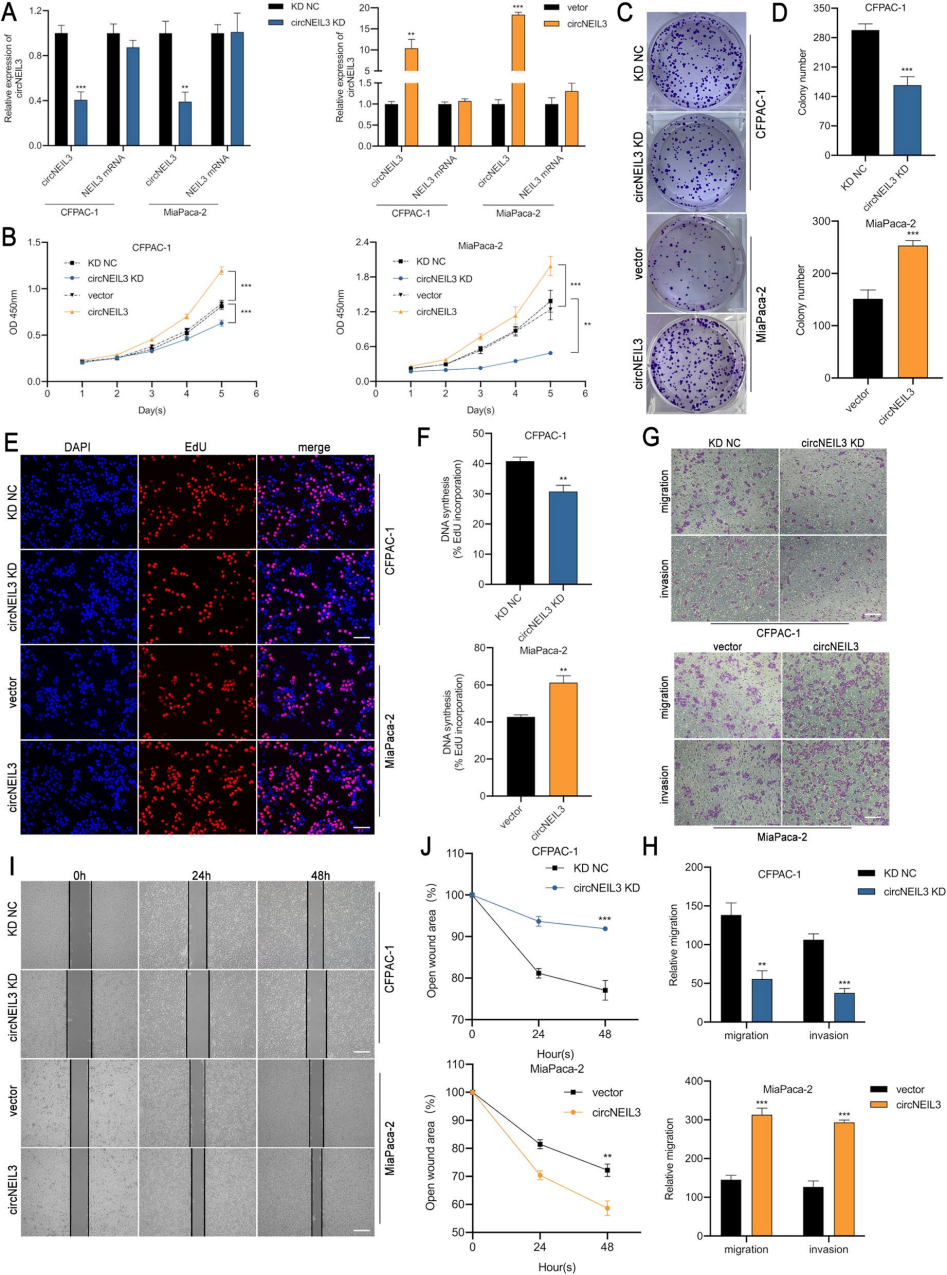
CircNEIL3 promotes the proliferation, migration and invasion of PDAC cells in vitro. **a.** RT-qPCR analysis of circNEIL3 and NEIL3 mRNA expression in CFPAC-1 and MiaPaca-2 cells transfected with a lentivirus and circNEIL3 plasmid. **b.** The growth curves of cells were evaluated by CCK-8 assays after knocking down and overexpressing circNEIL3 in CFPAC-1 and MiaPaca-2 cells. **c-d.** Colony formation assays were performed to evaluate cell proliferation. **e-f.** EdU assays of PDAC cells was performed to evaluate cell proliferation. The samples were imaged at 200× magnification. Scale bar = 50 μm. **g-h**. Transwell assays were performed to assess the migration and invasion abilities of PDAC cells. The samples were imaged at 100× magnification. Scale bar = 100 μm. **i-j.** Cell migration was assessed using a wound healing assay. The samples were imaged at 100× magnification. Scale bar = 100 μm. All data are presented as the means ± SD of three independent experiments. *p < 0.05, **p < 0.01, ***p < 0.001

The correction does not affect the conclusion or discussion of this article.

## Supplementary Information

**Additional file 1: Figure S3**. ADAR1 downregulation reverses the oncogenic phenotype induced by circNEIL3 overexpression. **a-i**. CCK-8, colony formation, EdU, transwell and wound healing assay results showed that transfection with the ADAR1 shRNA inhibited the proliferation, migration, and invasion abilities of MiaPaca-2 cells, which was reversed after cotransfection with the circNEIL3 plasmid. The EdU samples were imaged at 200× magnification. Scale bar = 50 μm. The transwell and hound healing samples were imaged at 100× magnification. Scale bar = 100 μm. All data are presented as the means ± SD of three independent experiments. *p < 0.05, **p < 0.01, ***p < 0.001, ****p < 0.0001.
